# GLUT3 drives paclitaxel resistance in peritoneal metastatic gastric cancer by promoting H3K18 lactylation-mediated MAPKAP1 transcription to suppress ferroptosis

**DOI:** 10.7150/ijbs.130059

**Published:** 2026-04-23

**Authors:** Ying Sun, Xirui Duan, Benyan Zhang, Cheng Xiong, Jun Ji, Qu Cai, Wang Yao, Jinling Jiang, Junwei Wu, Chao Wang, Liting Guo, Chenfei Zhou, Beiqin Yu, Feng Qi, Jun Zhang

**Affiliations:** 1 Department of Oncology, Ruijin Hospital, Shanghai Jiao Tong University School of Medicine, Shanghai Key Laboratory of Gastric Neoplasms, Shanghai, 200025, China.; 2 Department of Pathology, Ruijin Hospital, Shanghai Jiao Tong University School of Medicine, Shanghai, 200025, China.; 3 Department of General Surgery, Shanghai Key Laboratory of Gastric Neoplasms, Shanghai Institute of Digestive Surgery, Ruijin Hospital, Shanghai Jiao Tong University School of Medicine, Shanghai, 200025, China.

**Keywords:** gastric cancer, paclitaxel resistance, ferroptosis, glucose transporter 3, histone lactylation

## Abstract

**Background:**

Paclitaxel-based intraperitoneal chemotherapy (IPC) is a cornerstone strategy for treating gastric cancer peritoneal metastasis (GCPM). However, a subset of patients exhibit resistance to this therapy. Our study revealed that glucose transporter type 3 (GLUT3) is a key mediator of paclitaxel resistance in GCPM, although its precise mechanism of action remains to be fully elucidated.

**Methods:**

Single-cell (nucleus) sequencing and immunohistochemical staining were used to analyze paclitaxel-resistant and paclitaxel-sensitive GCPM tissue samples. GLUT3 was knocked down in AGS and HGC27 cells and overexpressed in MKN45 cells to establish the corresponding experimental models. The CUT&Tag and ChIP-qPCR techniques were utilized to elucidate the GLUT3-histone H3 lysine 18 lactylation (H3K18la)-mitogen-activated protein kinase associated protein 1 (MAPKAP1) regulatory axis. A mouse peritoneal metastasis model was used to evaluate the ability of GLUT3 targeting to reverse ferroptosis resistance and paclitaxel chemoresistance.

Results: GLUT3, glutathione peroxidase 4 (GPX4), and solute carrier family 7 member 11 (SLC7A11) expression was significantly upregulated in paclitaxel-resistant GCPM tissues. Elevated GLUT3 expression correlated with poor prognosis in GC patients. Functionally, GLUT3 knockdown sensitized GC cells to both Erastin and paclitaxel, whereas GLUT3 overexpression conferred therapeutic resistance. Mechanistically, GLUT3 upregulated hexokinase 3 (HK3) expression, increasing glucose-6-phosphate (G6P) and lactate production. Elevated lactate levels supported E1A binding protein p300 (p300)-mediated H3K18la enrichment at the MAPKAP1 promoter, thereby activating its transcription. Rescue assays indicated that depletion of MAPKAP1 restored ferroptosis sensitivity in GC cells. *In vivo*, compared with paclitaxel monotherapy, the combination of GLUT3 inhibition and paclitaxel not only reduced tumor weight by 75.47% (*P<0.05*) but also significantly suppressed the expression of MAPKAP1, GPX4, and SLC7A11.

**Conclusion:**

Targeting GLUT3-H3K18la-MAPKAP1 reverses paclitaxel resistance by inducing ferroptosis, providing a novel combination strategy for treating refractory GCPM.

## 1. Introduction

Peritoneal metastasis of gastric cancer (GCPM) represents the most aggressive metastatic form of advanced gastric cancer, occurring in approximately 40%-60% of patients, with a median overall survival (OS) typically ranging from only 3-6 months^1^. Paclitaxel is a key second-line therapeutic agent recommended by national and international guidelines following failure of first-line treatment. Recent clinical studies have demonstrated that the combined intraperitoneal and intravenous paclitaxel plus S-1 (NIPS) regimen significantly improves survival in GCPM patients, increasing the median OS to 19.4 months (compared with 13.9 months in the traditional intravenous paclitaxel plus oral S-1 group; HR=0.66; *P=0.005*). The 1-year and 2-year OS rates reached 69.6% and 37.2%, respectively (compared with 54.1% and 20.3% in the control group)^2^. However, resistance to paclitaxel still develops in a subset of patients receiving the NIPS regimen. Therefore, elucidating the mechanisms underlying paclitaxel resistance and identifying novel therapeutic targets to sensitize GCPM to paclitaxel are crucial for further increasing the efficacy of this effective combination regimen or overcoming its limitations.

Ferroptosis is a novel iron-dependent form of regulated cell death characterized by oxidative stress, iron accumulation, and excessive production of lipid reactive oxygen species (ROS). Studies indicate that targeting ferroptosis can reverse drug resistance in various tumors^3^,^4^. Consequently, clarifying the regulatory mechanisms of ferroptosis, including imbalances in redox homeostasis, iron metabolism, and lipid metabolism, is vital for improving cancer treatment. A detailed understanding of the molecular mechanisms of ferroptosis in the context of paclitaxel resistance has significant scientific and clinical value for overcoming resistance and increasing therapeutic efficacy. Previous studies have revealed that in triple-negative breast cancer, the OTU domain-containing protein 5 (OTUD5) suppresses ferroptosis by stabilizing the solute carrier family 7 member 11 (SLC7A11) protein, mediating paclitaxel resistance^5^. Collectively, these findings demonstrate that ferroptosis resistance is a key mechanism underlying paclitaxel resistance^5^-^7^. However, the specific molecular mechanisms of ferroptosis resistance in paclitaxel-resistant GCPM remain unclear.

To elucidate the molecular mechanisms underlying ferroptosis resistance in patients with paclitaxel-resistant GCPM, we focused on a core hallmark of cancer, namely, glucose metabolic reprogramming, also known as the Warburg effect^8^,^9^, which is a critical factor that regulates cellular ferroptosis and chemotherapy resistance^10^,^11^. Notably, lactate, the end product of glycolysis, is not merely a metabolic waste product. Its carboxyl group can form an amide bond with the ε-amino group of lysine (Lys) residues on proteins, driving protein lactylation modification. Mounting evidence indicates that this lactylation modification plays a pivotal role in regulating cellular ferroptosis and drug resistance. It has been reported that histone lactylation suppresses ferroptosis by upregulating the expression of the glutamate-cysteine ligase catalytic subunit (GCLC), thereby promoting oxaliplatin resistance in colorectal cancer stem cells^12^. Therefore, lactylation serves as a key link between cellular metabolism and ferroptosis resistance. Ferroptosis resistance mediated by this process constitutes an important mechanism of chemotherapy resistance in tumors. Nevertheless, the specific molecular mechanisms by which glycolytic metabolic reprogramming activates ferroptosis resistance, leading to paclitaxel resistance in GCPM, remain to be fully elucidated.

In this study, we demonstrated that glucose transporter type 3 (GLUT3) drives hexokinase 3 (HK3)-mediated glucose-6-phosphate (G6P) production, increasing glycolytic flux and lactate accumulation. Elevated lactate levels supported E1A binding protein p300 (EP300)-mediated histone H3 lysine 18 lactylation (H3K18la), which upregulated mitogen-activated protein kinase associated protein 1 (MAPKAP1) transcription. MAPKAP1, a key ferroptosis resistance effector, mediated ferroptosis resistance and paclitaxel unresponsiveness in GC cells. These findings suggest that GLUT3 is a therapeutic target for sensitizing GC cells to ferroptosis, suggesting a promising strategy for treating GCPM.

## 2. Materials and Methods

### 2.1 Data sources and bioinformatics analysis

Publicly available data were acquired from established repositories: RNA sequencing profiles of 375 gastric adenocarcinomas (TCGA-STAD cohort) and microarray data from GSE54129 (111 tumors/21 normal samples) were accessed through the Xena Browser and the Gene Expression Omnibus (GEO), respectively. Plasma metabolic data from 702 individuals (389 gastric cancer patients/313 controls) with clinical TNM staging data were obtained from supplementary files of a published study (PMC10891053). Key metabolic pathways, including the tricarboxylic acid (TCA) cycle, glycolysis/gluconeogenesis, and downstream signaling of GLUT3, were mapped to reference pathways in WikiPathways (WP534) and KEGG (hsa00010). Genome-wide CUT&Tag assays were performed by Shanghai Jiayin Biotechnology Ltd (Shanghai, China). The list of genes associated with ferroptosis was obtained from FerrDb (http://www.zhounan.org/ferrdb), a curated database for ferroptosis regulators and markers.

Single-nucleus suspensions were prepared from clinical pathological tissue sections obtained from 2 gastric cancer patients with paclitaxel-resistant peritoneal metastasis and 2 with paclitaxel-sensitive peritoneal metastasis. Single-nucleus suspensions were converted to barcoded snRNA-seq libraries using the Chromium Single Cell 3' Library, Gel Bead & Multiplex Kit and Chip Kit (10x Genomics) according to the manufacturer's instructions. Based on canonical markers, major cell lineages were annotated as follows: Tumor cells (EPCAM, KRT18, KRT19), T/NK cells (PTPRC, CD3D, CD3E, CD3G, TRAC, NKG7), B cells (PTPRC, MS4A1, CD79A, CD79B, IGHM), Myeloid cells (PTPRC, CD68), Fibroblasts (MMP2, COL1A2, DCN), Endothelial cells (PLVAP, PTPRB), and Smooth muscle cells (ADIRF, RERGL). Differentially expressed genes (DEGs) between paclitaxel-resistant and paclitaxel-sensitive groups were identified using the Seurat package, with a threshold of |Avg_logFC| > 0.25 and P_val < 0.01.

### 2.2 Human tissue samples

Human tissue samples from multiple sources were utilized in this study. Retrospective specimens consisted of formalin-fixed, paraffin-embedded (FFPE) tissue sections archived in the Department of Pathology, Ruijin Hospital, Shanghai Jiao Tong University School of Medicine. This cohort included three GC samples: one paclitaxel-sensitive GCPM lesion, one paclitaxel-resistant GCPM lesion, and one primary GC lesion. Additionally, four pathological tissue sections of GCPM samples (two paclitaxel-sensitive and two paclitaxel-resistant GCPM lesions) were collected and subjected to snRNA-seq analysis. For the tissue microarray component, two commercial gastric cancer tissue microarrays (Shanghai Outdo Biotech Company) were used: Array HStmA180Su19 contained 94 spots of primary gastric cancer tissues and 86 spots of normal gastric control tissues, and Array HStm-Ade180Sur-06 contained 90 spots of primary gastric cancer tissues and 90 spots of normal gastric control tissues.

### 2.3 Cell culture and reagents

Human GC cell lines (AGS, HGC27, MKN45, MKN74, NCI-N87 and NUGC-4) and a normal gastric mucosal cell line (GES-1) were purchased from the cell bank of the Chinese Academy of Science (Shanghai, China). These cells were cultured in Dulbecco's modified Eagle's medium (11965092; Gibco) supplemented with 10% fetal bovine serum (10270-106; Gibco) and 1% penicillin‒streptomycin (15140-122; Gibco). All the cells were maintained at 37°C in a humidified incubator with 5% CO_2_. All the experimental reagents used are listed in Supplementary Table S3.

### 2.4 Plasmids and lentiviral transfection

Lentiviral-based small hairpin RNA (shRNA) to silence GLUT3 (5'-TGTCACTGGTGGCTGCTTTAT-3'), GLUT3 overexpression plasmid, MAPKAP1 overexpression plasmid and their corresponding control lentivirus/plasmids were constructed by Genomeditech (Shanghai, China). All lentivirus transfections were performed according to the manufacturer's instructions, and stably infected cell lines were selected using 10 μg/mL puromycin for at least two weeks.

These plasmids were transduced into GC cells with NanoTrans™ Transfection Reagent 3000 (CT0006; KGI Biotechnology, China). Briefly, 5 μL of NanoTrans™ Enhancer Reagent and 2.5 μg of plasmid DNA were diluted in 125 μL of Opti-MEM^®^ Reduced Serum Medium, while 5 μL of NanoTrans™ Transfection Reagent 3000 was separately diluted in 125 μL of Opti-MEM^®^. The two solutions were combined and incubated at room temperature for 15 min. The resulting complex mixture was added dropwise to the cells and incubated for 6 h at 37°C with 5% CO₂. The medium was replaced with fresh complete medium 6 h post-transfection, and puromycin selection (10 μg/mL) was initiated 72 h post-transfection.

### 2.5 Small interfering RNA (siRNA) transfection

siRNAs specifically targeting MAPKAP1/EP300 were obtained from Genomeditech. The transfection of siRNAs into GC cells was performed with NanoTrans™ Transfection Reagent 3000 (CT0006; KGI Biotechnology, China) according to the manufacturer's instructions. The siRNA sequences are listed in Supplementary Table S4.

### 2.6 Immunohistochemical (IHC) staining

The IHC procedure was performed as described previously^13^. IHC staining quantification was conducted with a histological scoring (H-score) approach. The staining intensity was graded as follows: 0 (negative), 1 (weak), 2 (intermediate), or 3 (strong). The proportions of positively stained cells were categorized as follows: 1 (0-25%), 2 (26-50%), 3 (51-75%), or 4 (76-100%). The final IHC score was calculated as the staining intensity score × the percentage of target protein-positive cells. The median H-scores were used as cutoff to separate patients into low and high expression subgroups. Two independent pathologists who were blinded to the clinical data performed all the evaluations. The antibodies used for IHC staining are listed in Supplementary Table S5.

### 2.7 Western blotting analysis

For Western blotting, whole-cell extracts were lysed for 30 min in RIPA lysis buffer (20-188, Millipore Sigma) containing 1% protease/phosphatase inhibitor cocktail, followed by sonication and centrifugation at 12,000 rpm for 10 min. The supernatant was mixed with 5× loading buffer at a 4:1 volume ratio (4 volume sample + 1 volumes of 5× loading buffer). Protein samples were boiled at 100°C for 10 min, separated by SDS‒polyacrylamide gel electrophoresis (SDS‒PAGE), and transferred to PVDF membranes. The target proteins were visualized by chemiluminescence after incubation with primary and secondary antibodies. Grayscale analysis was performed using ImageJ software, with β-actin serving as the internal reference for normalization. The antibodies used for Western blotting are listed in Supplementary Table S5.

### 2.8 RNA extraction and quantitative real-time polymerase chain reaction (qRT‒PCR)

Total RNA was extracted using TRIzol reagent (Invitrogen Life Technologies) according to the manufacturer's protocol. 1 μg of total RNA were reverse transcribed to cDNA using an RT reagent Kit with gDNA Eraser (RR047, Takara, Japan). Quantitative PCR (qPCR) was performed using SYBR Green Master Mix (11202ES, Yeasen) and primers listed in Table S6 on an ABI 7500 Real-Time PCR system. β-actin was used as the endogenous reference gene. The key primer sequences used in this study are as follows: MAPKAP1 forward primer: 5'-GGTGGACACCGATTTCCCC-3', reverse primer: 5'-CGCTTCACTGCCTTCAGTAAGA-3'; GLUT3 forward primer: 5'-GCTGGGCATCGTTGTTGGA-3', reverse primer: 5'-GCACTTTGTAGGATAGCAGGAAG-3'; internal reference gene β-actin forward primer: 5'-CATGTACGTTGCTATCCAGGC-3', reverse primer: 5'-CTCCTTAATGTCACGCACGAT-3'. Relative mRNA expression levels were calculated using the 2^(-ΔΔCt) method.

### 2.9 Cell proliferation and colony formation assays

1) For Counting Kit-8 (CCK8) analysis, stable GLUT3-overexpressing and GLUT3-knockdown GC cells were seeded into a 96-well plate at approximately 1,000 cells/well in 100 μL of medium. In accordance with the protocol of the CCK8 assay kit (CK04, Dojindo, Kumamoto, Japan), 100 μL complete medium containing 10 μL CCK8 reagent was added to each well at different time points (0, 24, 48, 72, 96 and 120 h) to replace the original medium. After the plates were incubated in the dark at 37°C for 2 h, we measured the absorbance at a wavelength of 450 nm to calculate cell viability.

2) For colony formation assays, cells were seeded in a 6-well plate (1000 cells/well) and cultured at 37°C and 5% CO_2_. After 15 days, the cells were fixed with 4% paraformaldehyde and then stained with 0.1% crystal violet. The number of colonies was determined using ImageJ software (National Institutes of Health, Bethesda, MD, USA).

### 2.10 Transwell migration and invasion experiments

Cell migration and invasion were assessed with Transwell chambers. For invasion assays, the upper chambers were precoated with Matrigel (BD Biosciences), whereas uncoated chambers were used for migration assays. Briefly, 5×10⁴ cells in serum-free medium were seeded into the upper chambers, and medium supplemented with 10% FBS was added to the lower chambers as a chemoattractant. After 24 h of incubation, the cells that migrated/invaded to the lower membrane surface were fixed with 4% paraformaldehyde for 30 min, stained with 0.1% crystal violet for 30 min. Cell numbers were quantified by counting three random fields per chamber.

### 2.11 Wound healing assay

Cell migration was analyzed with a Culture-Insert 2 Well (80209; ibidi, Gräfelfing, Germany). Cells were seeded at 3×10⁴ cells per chamber and cultured to 100% confluence (24 h). After the silicone insert was removed to create a standardized 500-μm wound, the wells were washed twice with PBS and replenished with low-serum medium (1% FBS). Images at identical locations were captured at 0, 24 and 48 h with an inverted microscope.

### 2.12 Cell viability assay

Cell viability was assessed with a CCK8 kit (CK04; Dojindo, Kumamoto, Japan). Cells were seeded in 96-well plates at 5×10³ cells/well. After 24 h, the cells were treated with the following agents: paclitaxel (HY-B0015; MedChemExpress) at 25, 50, 100, 150, and 250 nM (AGS/HGC27); 2, 4, 6, 8, 10 μM (MKN45); Erastin (HY-15763; MedChemExpress) at 2.5, 5, 7.5, 10, and 20 μM; and ferrostatin-1 (Fer-1) (HY-100579; MedChemExpress) at 2 μM; and GLUT inhibitor-1 (HY-139605; MedChemExpress) at 1 μM for 24 h. Subsequently, 10 μL of CCK8 reagent was added per well, and the cells were incubated for 2 h at 37°C. We measured the absorbance at a wavelength of 450 nm to calculate cell viability.

### 2.13 Measurement of total ROS, lipid peroxidation, and MDA levels and the GSH/GSSG ratio

1) Total ROS measurement: According to the reactive oxygen species assay kit (CA1410; Solarbio), the DCFH-DA stock solution was diluted with serum-free medium at a 1:1000 ratio to prepare a 10 μM working solution. The cells were incubated with this working solution at 37°C in the dark for 45 min. After the cells were washed with PBS, the fluorescence intensity was quantified with either a flow cytometer or a fluorescence microscope at an excitation/emission wavelength (Ex/Em) of 488/525 nm.

2) Lipid peroxidation: C11-BODIPY⁵⁸¹/⁵⁹¹ (D3861; Thermo Fisher) stock solution (10 mM in DMSO) was diluted 1:1000 in complete medium supplemented with 10% FBS to prepare a 10 μM working solution. The cells were incubated with this working solution at 37°C in the dark for 45 min. After the cells were washed with PBS, the oxidized state and reduced state fluorescence signals were analyzed by flow cytometry.

3) MDA levels: Intracellular MDA levels were quantified with a Lipid Peroxidation MDA Assay Kit (S0131S; Beyotime). Cells were lysed on ice for 30 min in ice-cold Western and IP Cell Lysis Buffer (P0013; Beyotime) at a concentration of 1×10⁶ cells/100 μL. The lysates were then centrifuged at 12,000×g for 10 min at 4°C. 5 μL of the supernatant was taken to detect the protein content. One hundred microliters of the supernatant was mixed with 200 μL of MDA working solution. The mixture was heated at 100°C for 15 min. After water bath cooling to room temperature, the samples were centrifuged at 1000×g for 10 min at room temperature to remove the precipitates. The supernatant was collected, and its absorbance was measured at 532 nm with a microplate reader. The MDA concentration was calculated on the basis of a standard curve generated from standards and normalized to the total protein concentration determined by the BCA method.

4) GSH/GSSG ratio: Intracellular GSH/GSSG levels were quantified with the Glutathione Assay Kit (S0053; Beyotime). Total glutathione detection: After the cells were washed with PBS, the pellets were collected by centrifugation, and the supernatant was completely removed. Protein removal reagent M solution was added at 3 times the pellet volume, followed by thorough vortex mixing. The samples underwent two freeze‒thaw cycles alternating between liquid nitrogen and a 37°C water bath and then were incubated on ice for 5 min. After centrifugation at 10,000×g for 10 min at 4°C, the supernatant was collected for analysis. Subsequently, 10 μL of processed sample was mixed with 150 μL of total glutathione detection working solution and incubated at 25°C for 5 min. Then, 50 μL of 0.5 mg/mL NADPH was added. After mixing, the samples were reacted at 25°C for 25 min, after which the absorbance was measured at 412 nm. GSSG detection: Diluted GSH-scavenging auxiliary solution was added to the supernatant at a ratio of 100 μL sample:20 μL reagent and immediately vortexed. GSH-scavenging working solution was then added at a ratio of 100 μL of sample:4 μL of working solution, followed by immediate vortexing and reaction at 25°C for 60 min. Subsequently, 10 μL of processed sample was mixed with 150 μL of total glutathione detection working solution and incubated at 25°C for 5 min. Then, 50 μL of 0.5 mg/mL NADPH was added. After mixing, the samples were reacted at 25°C for 25 min, after which the absorbance was measured at 412 nm. GSH and GSSG concentrations were calculated with a standard curve, and the ratio was determined as follows: GSH/GSSG = [Total Glutathione - 2 × GSSG] / GSSG.

### 2.14 Transmission electron microscopy (TEM)

The cells were plated in 10 cm dish with a density of 6 × 10^5^ cells per dish and incubated overnight. Next, cells were treated with or without Erastin (10 μM). After treatment of 24 h, cells were collected and fixed with 2.5% glutaraldehyde at 4°C, followed by treatment with a 1% osmium tetraoxide for 2 h at room temperature. Subsequently, the sample were dehydrated in gradual ethanol and embedded in epoxy resin. Representative images were captured by TEM (Tecnai G2 F20 S-TWIN, FEI, USA) after slicing and staining with uranyl acetate and lead citrate.

### 2.15 Quantification of intracellular lactate levels

Intracellular lactate levels were quantified with a Lactate Assay Kit (BC2235; Solarbio). Briefly, approximately 5 × 10⁶ cells were resuspended in 1 mL of Extraction Buffer I and lysed by sonication on ice. The lysate was subsequently centrifuged at 12,000×g for 10 min at 4°C. Afterward, 0.8 mL of the supernatant was carefully mixed with 0.15 mL of Extraction Buffer II by gentle pipetting, followed by centrifugation at 12,000×g for 10 min at 4°C. The resulting supernatant was collected as the test sample. For the assay, 10 μL of this supernatant was mixed sequentially with 40 μL of Reagent 1, 10 μL of Reagent 2, and 20 μL of Reagent 4. After vortexing, the mixture was incubated at 37°C for 20 min. Then, 6 μL of Reagent 5 and 60 μL of Reagent 3 were added, and the reaction was incubated at 37°C for 20 min in the dark. After centrifugation at 10,000×g for 10 min at 25°C, the supernatant was discarded, and the pellet was dissolved in 200 μL of ethanol. The absorbance was measured at 570 nm. The lactate concentration (x) was determined from a lactate standard curve. The intracellular L-lactate content was calculated as follows: L-lactate (μmol/10⁶ cells) = x × (volume of supernatant + volume of Extraction Buffer II) ÷ (N × volume of supernatant ÷ volume of Extraction Buffer I) = 1.1875 × x ÷ N (N: cell count).

### 2.16 ChIP‒quantitative polymerase chain reaction (ChIP‒qPCR)

Chromatin immunoprecipitation (ChIP) was performed with an EZ-ChIP™ Kit (17-371; Millipore Sigma) with the following workflow: The cells were cross-linked with 1% formaldehyde for 10 min at room temperature (25°C), followed by quenching with 125 mM glycine. After lysis, the chromatin was fragmented to 200-500 bp via sonication. The lysates were precleared with Protein G Agarose and subsequently immunoprecipitated overnight at 4°C with 5 μg of a rabbit anti-H3K18la antibody (PTM-1427RM; PTM Bio) or a species-matched IgG control. Immune complexes were captured with Protein G Agarose and sequentially washed twice with low-salt buffer, twice with high-salt buffer, once with LiCl buffer, and once with TE buffer. The complexes were eluted with elution buffer, followed by cross-link reversal at 65°C for 4 h and digestion with 0.2 mg/mL proteinase K at 55°C for 2 h. DNA was subsequently purified with the spin columns of the kit. Purified DNA was analyzed by qPCR with SYBR Green Master Mix with target-specific primers. The ChIP-qPCR primers used in this study target the MAPKAP1 promoter region 7, and their sequences are as follows: forward primer: 5'-CGAACTAAGGGCTTTTCTCCGT-3', reverse primer: 5'-CCGAGCAGCAGCCCTATTAC-3'. The sequences of other primers are detailed in Table S6. Input control samples (representing 1% chromatin lysate, processed in parallel with IP samples through cross-link reversal, digestion, and purification) were used for normalization, with the results expressed as the fold enrichment.

### 2.17 Animal experiments evaluating GLUT3 function and therapeutic efficacy

To elucidate the role of GLUT3 in peritoneal metastasis and validate whether the combination of GLUT inhibitor-1 (HY-139605; MedChemExpress) and paclitaxel increases antitumor activity by promoting ferroptosis, two independent animal experiments were conducted. All experimental procedures strictly adhered to animal welfare and ethical guidelines and were approved by the Animal Ethics Committee of Ruijin Hospital, Shanghai Jiao Tong University School of Medicine (RJ2023034).

### 2.17.1 Assessment of the effect of GLUT3 overexpression on peritoneal metastasis

To evaluate the role of GLUT3 in peritoneal metastasis, the human gastric cancer cell line MKN45 was used. MKN45 cells stably transfected with an empty vector (MKN45-Vector) and MKN45 cells overexpressing GLUT3 (MKN45-GLUT3) were separately resuspended in sterile physiological saline. Each 5-6-week-old male nude mouse (BALB/c background) received an intraperitoneal injection of 200 μL of cell suspension containing 1×10⁷ cells. The mice were randomly and blindly assigned to two groups: the MKN45-Vector group and the MKN45-GLUT3 group. On day 21 after cell injection, *in vivo* fluorescence imaging (IVIS) was performed to capture abdominal fluorescence signals and assess tumor burden. The mice were then euthanized, the abdominal cavity was exposed, all the tumor nodules were collected, and the tumor weights were measured and photographed.

### 2.17.2 Evaluation of combined therapy targeting GLUT3-overexpressing peritoneal tumors

To validate whether the combination of GLUT inhibitor-1 and paclitaxel increase antitumor activity via the promotion of ferroptosis in GLUT3-overexpressing tumors, MKN45-GLUT3 cells were used. Twelve 5-6-week-old male nude mice (in the BALB/c background) each received an intraperitoneal injection of 200 μL of a suspension containing 1×10⁷ MKN45-GLUT3 cells. After successful model establishment, the tumor-bearing mice were randomly and blindly divided into four groups (n=3 per group) and treated via intraperitoneal injection every other day from day 22 to day 34 (7 administrations total). The groups and treatment regimens were as follows: saline group, paclitaxel group (15 mg/kg), GLUT inhibitor-1 group (10 mg/kg), and combination group. At the end of the treatment period, an IVIS was performed to assess tumor growth. The mice were then euthanized, the abdominal cavity was exposed, all the tumor nodules were collected, and the tumor weights were measured and photographed. Finally, the tumor tissues were sectioned for IHC staining of MAPKAP1, GPX4, and SLC7A11, and the intratumoral levels of MDA and the GSH/GSSG ratio were determined.

### 2.18 *In vivo* luminescence imaging

Prior to imaging, the mice were anesthetized with 2% isoflurane in oxygen. Under anesthesia, each nude mouse received an intraperitoneal injection of D-luciferin sodium salt (40901ES01; Yeasen Biotechnology, China). Bioluminescence imaging was performed 15 min post-injection with the IVIS Lumina LT system (Caliper Life Sciences, USA). The luciferase signal, which is indicative of the extent of peritoneal metastasis, was quantified by measuring total flux (photons/second) within a defined region of interest (ROI) encompassing the abdominal cavity/tumor sites with IVIS platform software. Image processing and data acquisition were conducted with Living Image software (version 4.3; Caliper Life Sciences, USA).

### 2.19 Tumor tissue grinding and assessments of MDA levels and the GSH/GSSG ratio

Rapidly frozen tumor tissues were weighed, ground into powder in liquid nitrogen, and transferred to prechilled centrifuge tubes. For GSH/GSSG detection, 10 mg of tissue powder was precisely weighed and vortexed with 30 μL of Protein Removal Reagent M. Subsequently, 70 μL of Reagent M was added, and the mixture was thoroughly homogenized with a glass homogenizer. The homogenate was incubated at 4°C for 10 min, followed by centrifugation at 10,000×g (4°C, 10 min). The supernatant was collected for quantitative analysis of GSH and GSSG levels according to the manufacturer's protocol for the Glutathione Assay Kit (S0053; Beyotime). For MDA detection, tissues were lysed with western and IP lysis buffer (P0013; Beyotime) at a 10% mass-to-volume ratio and maintained on ice throughout the process. The lysate was centrifuged at 12,000×g for 10 min, and the supernatant was subjected to MDA quantification following the protocol of the Lipid Peroxidation MDA Assay Kit (S0131S, Beyotime).

### 2.20 Statistical analysis

All experiments were performed with at least three independent biological replicates and a minimum sample size of 3 per group. Continuous data with a normal distribution are expressed as the mean ± standard deviation (SD). Statistical analyses were conducted with IBM SPSS Statistics v27.0 (IBM Corp., Armonk, NY, USA) and GraphPad Prism v9.0 (GraphPad Software, San Diego, CA, USA). Differences between two groups were assessed by unpaired Student's t test, whereas one-way ANOVA with Tukey's post hoc test was used for multigroup comparisons under a completely randomized design. Categorical data were analyzed by Pearson's χ² test (or Fisher's exact test when expected frequencies <5). Overall survival (OS) was estimated via the Kaplan‒Meier method, with statistical significance evaluated by the log-rank test. A two-tailed *P* < 0.05 was considered to indicate statistical significance.

## 3. Results

### 3.1 Coexpression of GLUT3 and ferroptosis-related genes in paclitaxel-resistant GCPM tissues

**Figure 1:**
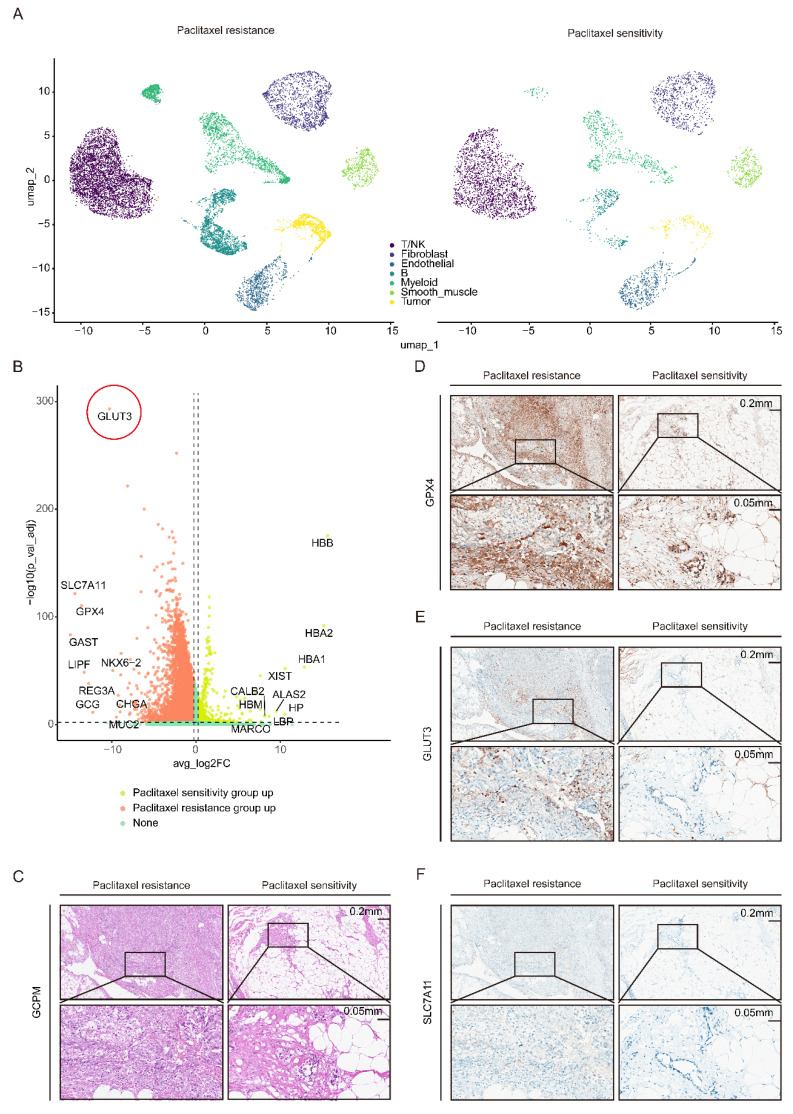
** GLUT3 and the ferroptosis resistance genes GPX4 and SLC7A11 are upregulated in paclitaxel-resistant GCPM tissues.** A) UMAP plot of the single-cell (nucleus) sequencing data for seven main cell types, namely, T/NK cells, B cells, fibroblasts, myeloid cells, endothelial cells, tumor cells, and smooth muscle cells. B) Volcano map showing the differences in the expression of genes in gastric cancer peritoneal metastasis tissues with paclitaxel resistance or sensitivity. C-F) Representative IHC staining for GLUT3, GPX4, and SLC7A11 and H&E staining of gastric cancer peritoneal metastasis tissues with paclitaxel resistance or sensitivity. Scale bars: 200 μm and 50 μm. Immunohistochemical staining was quantified using the H-score, calculated as follows: H-score = staining intensity (0-3) × percentage of positive cells (1-4). The staining intensity was graded as follows: 0 (negative), 1 (weak), 2 (intermediate), or 3 (strong). The proportions of positively stained cells were categorized as follows: 1 (0-25%), 2 (26-50%), 3 (51-75%), or 4 (76-100%). Abbreviations: DEGs: Differentially Expressed Genes; H&E: Hematoxylin and Eosin staining; IHC: Immunohistochemical.

To elucidate the cellular and molecular mechanisms underlying paclitaxel resistance in GCPM patients, we performed single-nucleus RNA sequencing (snRNA-seq) on pathological tissue sections of GCPM samples from two paclitaxel-resistant patients and two paclitaxel-sensitive patients, followed by data integration. Uniform Manifold Approximation and Projection (UMAP) dimensionality reduction and clustering analysis, combined with annotation using classical marker genes, revealed seven major cell subpopulations: T/NK cells, B cells, myeloid cells, fibroblasts, endothelial cells, smooth muscle cells, and tumor cells **(Figure 1A)**. Analysis of the tumor cell subcluster revealed significantly upregulated expression of GLUT3 and ferroptosis-related genes (GPX4 and SLC7A11) in tumor cells from the resistant group compared with those from the sensitive group **(Figure S1A-C)**. Volcano plot analysis further confirmed coordinated upregulation of GLUT3, GPX4, and SLC7A11 expression specifically within the paclitaxel-resistant group **(Figure 1B)**. Finally, immunohistochemical (IHC) staining of clinical GCPM specimens validated these snRNA-seq findings: the protein expression levels of GLUT3, GPX4, and SLC7A11 were markedly greater in paclitaxel-resistant tissue than in paclitaxel-sensitive tissues **(Figure 1C-F)**. Collectively, the concordant upregulation of GLUT3 and ferroptosis-inhibiting genes (GPX4 and SLC7A11) in paclitaxel-resistant GCPM tissue, validated at both the transcriptome and protein levels, implies a potential mechanistic link between glucose metabolism and ferroptosis suppression in paclitaxel-resistant GCPM.

### 3.2 GLUT3 is upregulated in gastric cancer and associated with a poor prognosis

**Figure 2:**
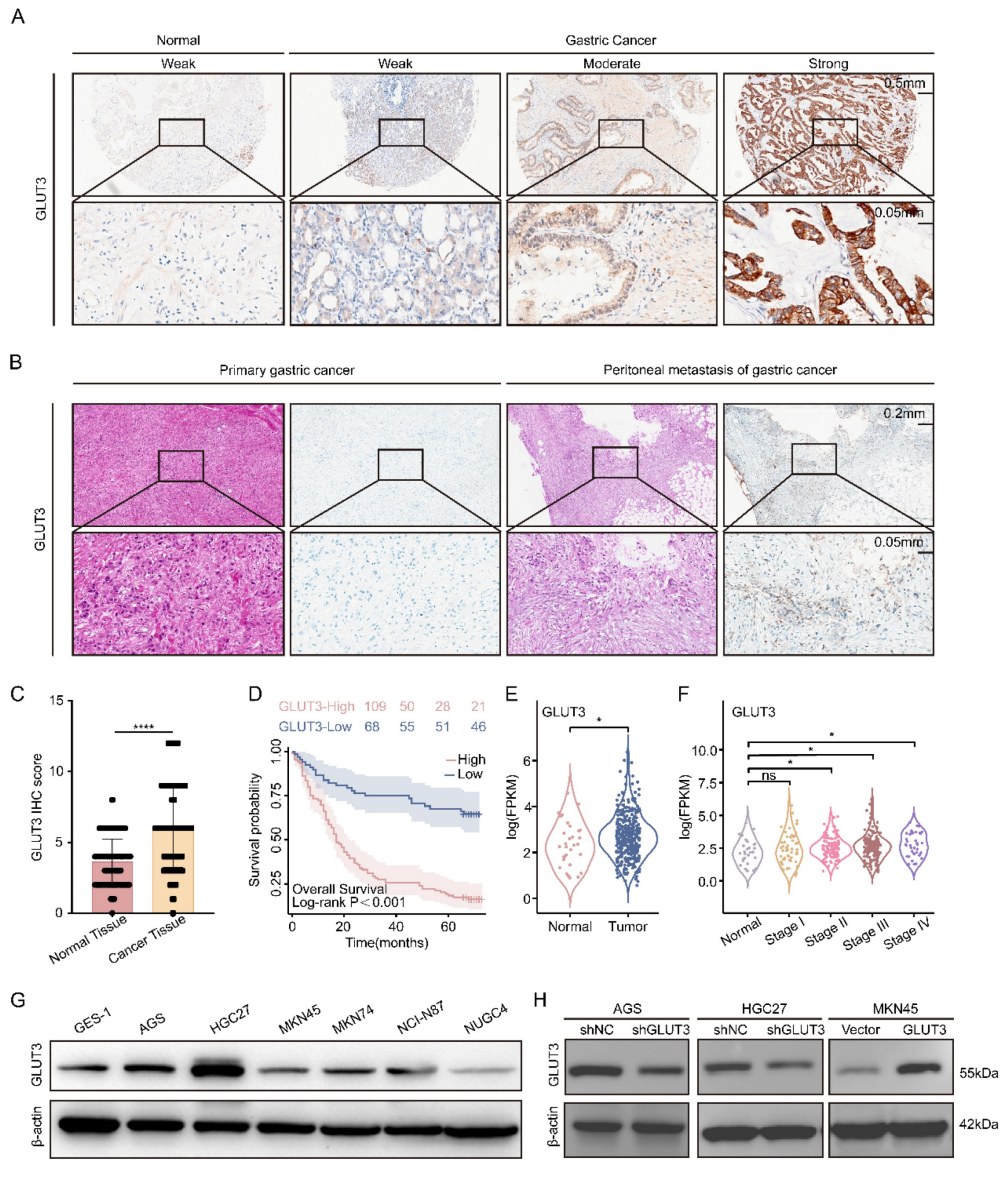
** GLUT3 is upregulated and associated with poor prognosis in patients with GC.** A) Representative GLUT3 IHC staining in a cohort comprising 86 paired gastric tumor/nontumor tissues and 8 unpaired gastric tumor tissues. Scale bars: 500 μm and 50 μm. B) Representative IHC staining for GLUT3 in paired primary gastric tumor and corresponding peritoneal metastasis tissues. Scale bars: 200 μm and 50 μm. C) Quantification of GLUT3 IHC staining scores in tumor and nontumor gastric tissues. D) Kaplan‒Meier curves for overall survival according to GLUT3 protein expression in GC specimens from tissue microarrays (n=177 cases). E) GLUT3 mRNA levels in tumor and normal tissues from patients with gastric cancer in the TCGA cohort. F) GLUT3 mRNA levels in stage I to IV gastric cancer tissues compared with those in normal tissues in the TCGA gastric cancer cohort. G) Protein baseline expression of GLUT3 in gastric cancer cells and normal gastric mucosal cells. H) The knockdown and overexpression efficiencies of GLUT3 were validated at protein level in GC cells. All the data are presented as the mean ± SD. *P<0.05, ****P<0.0001, ns: not significant. Immunohistochemical staining was quantified using the H-score, calculated as follows: H-score = staining intensity (0-3) × percentage of positive cells (1-4). The staining intensity was graded as follows: 0 (negative), 1 (weak), 2 (intermediate), or 3 (strong). The proportions of positively stained cells were categorized as follows: 1 (0-25%), 2 (26-50%), 3 (51-75%), or 4 (76-100%). Abbreviations: IHC: Immunohistochemical.

To systematically evaluate the clinical significance of the glucose transporter (GLUT) family in GC, we integrated public databases and tissue microarray (TMA) data. Analysis of the TCGA cohort revealed significantly elevated mRNA levels of GLUT1, GLUT3, GLUT6, GLUT8, GLUT9, GLUT10, and GLUT11 in GC tissues compared with normal tissues **(Figure S2A)**. Notably, GLUT3 expression was significantly higher in deceased patients, than in surviving patients, while no statistically significant differences between these groups were observed for the other GLUT family members analyzed **(Figure S2B)**. Survival analysis further revealed the prognostic value of GLUT3: its high expression was significantly associated with shortened OS and had the most pronounced negative effect. Although high GLUT2 expression was also correlated with poor prognosis, this statistical significance was lower than that of GLUT3. The remaining GLUT family members had no significant prognostic value **(Figure S2C-F, S3A-B)**. Further analysis of TCGA data demonstrated that GLUT3 expression was significantly higher in GC tissues than in normal tissues, with this difference being particularly significant in stage II to IV patients **(Figure 2E-F)**. This finding was validated in the GEO dataset: elevated mRNA expression of GLUT3, GLUT5, GLUT8, GLUT9, GLUT10, and GLUT11 was observed in GC tissues** (Figure S2G)**, and high GLUT3 expression similarly predicted significantly shorter OS **(Figure S2H)**. Additionally, TMA analysis confirmed significantly stronger GLUT3 expression in GC tissues than in paired adjacent normal tissues **(Figure 2A, 2C)**. Crucially, analysis of clinical paired samples revealed that compared with primary tumors, peritoneal metastatic lesions presented higher GLUT3 protein expression levels **(Figure 2B)**. Finally, within the TMA cohort of 184 patients (7 cases were excluded due to missing data), patients were stratified by median GLUT3 expression into high-expression (n=109) and low-expression (n=68) groups. Kaplan-Meier analysis revealed significantly shorter OS in patients with high GLUT3 expression (*P<0.001*)** (Figure 2D)**. GLUT3 expression levels were also significantly correlated with T stage and N stage **(Table S1)**. Multivariate Cox regression ultimately revealed high GLUT3 expression (hazard ratio (HR): 2.717; 95%CI: 1.689-4.372; *P<0.001*), T3-4 stage (HR: 2.906; 95%CI: 1.401-6.028; *P=0.004*), and N2-3 stage (HR: 3.219; 95%CI: 1.963-5.280; *P<0.001*) as independent risk factors for patient prognosis **(Table S2)**. Collectively, these multiplatform data consistently demonstrate that GLUT3 is aberrantly overexpressed in GC and that its high expression is significantly associated with poor prognosis, identifying GLUT3 as a promising prognostic biomarker for gastric cancer.

### 3.3 GLUT3 drives malignant progression in gastric cancer by promoting cell proliferation, migration, and invasion

To investigate the oncogenic role of GLUT3 in GC, we first assessed its endogenous expression levels across multiple GC cell lines **(Figure 2G)**. On the basis of high GLUT3 expression in AGS and HGC27 cells and relatively low expression in MKN45 cells, we performed knockdown (KD) of GLUT3 in AGS and HGC27 cells and overexpression (OE) of GLUT3 in MKN45 cells. Effective KD and OE were confirmed at both the mRNA and protein levels **(Figure 2H, S4A-B)**. We subsequently examined the effects of GLUT3 on GC cell growth, migration, and invasion. The results demonstrated that GLUT3 KD significantly suppressed the proliferation and colony formation capacities of AGS and HGC27 cells. Conversely, GLUT3 OE markedly increased these capacities in MKN45 cells** (Figure S5A-C)**. Similarly, GLUT3 KD significantly reduced the migration and invasion abilities of AGS and HGC27 cells, whereas GLUT3 OE significantly promoted migration and invasion in MKN45 cells **(Figure S5D-G)**. To validate the role of GLUT3 *in vivo*, we established a GCPM mouse model and found that GLUT3 OE indeed promoted peritoneal metastatic tumor growth **(Figure S5H-J)**. Collectively, these results indicate that GLUT3 drives malignant progression in gastric cancer by increasing the proliferative and metastatic potential of GC cells.

### 3.4 GLUT3 confers resistance to paclitaxel cytotoxicity in GC cells by suppressing ferroptosis

**Figure 3:**
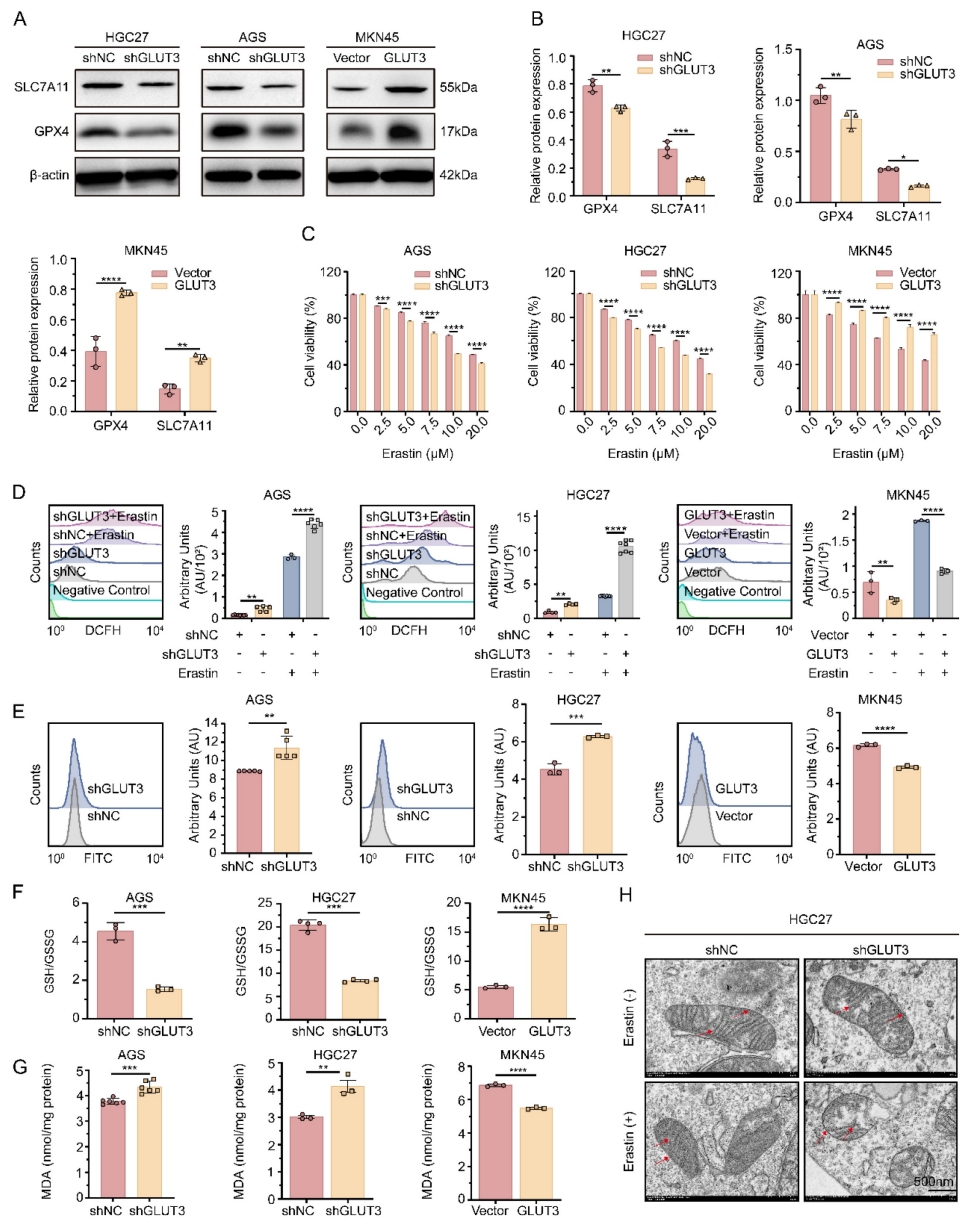
** GLUT3 deficiency promotes Erastin-induced ferroptosis in GC cells.** A-B) Western blot of GPX4 and SLC7A11 expression in AGS/shGLUT3, HGC27/shGLUT3 and MKN45/GLUT3 cells and their corresponding negative control cells. C) AGS/shGLUT3, HGC27/shGLUT3 and MKN45/GLUT3 cells and their negative control cells were treated with increasing concentrations of Erastin (0, 2.5, 5, 7.5, 10 and 20 µM) for 24 h, and the viability of the cells was measured using a CCK8 assay. D) The levels of total ROS in the indicated cells were determined by DCFH-DA staining and flow cytometry after treatment with DMSO or the Erastin (10 μM) for 24 h (n≥3). E) Levels of lipid ROS in the indicated cells were determined by C11-BODIPY staining and flow cytometry (n≥3). F) The ratio of GSH/GSSG in the indicated cells was measured by a microplate spectrophotometer (n≥3). G) The levels of MDA in the indicated cells were measured by a microplate spectrophotometer (n≥3). H) Transmission electron microscopy was conducted in HGC27/shGLUT3 cells and control cells after treatment with DMSO or Erastin (10 μM) for 24 h. Scale bar: 500 nm. The red arrows indicate disrupted mitochondrial cristae and increased density of mitochondrial membranes. All the data are presented as the mean ± SD. *P<0.05, **P <0.01, ***P<0.001, ****P<0.0001. Abbreviations: ROS: Reactive Oxygen Species; CCK8: Cell Counting Kit-8; MDA: Malondialdehyde; GSH: Glutathione (reduced); GSSG: Glutathione Disulfide (oxidized).

Given that GLUT3 is cataloged in the ferroptosis-related gene database FerrDb, although its specific role in ferroptosis, particularly in cancer, remains undefined, and that our snRNA-seq analysis revealed the high expression of ferroptosis-related genes (GPX4 and SLC7A11) in paclitaxel-resistant GCPM tissues** (Figure S1A-C)**, we hypothesized that GLUT3 plays a critical role in regulating ferroptosis in GC cells. We performed a series of experiments to test this hypothesis. Protein analysis demonstrated that GLUT3 KD significantly reduced the expression of GPX4 and SLC7A11 in AGS and HGC27 cells. Conversely, GLUT3 OE markedly increased the levels of these proteins in MKN45 cells **(Figure 3A-B)**. To investigate the functional impact of GLUT3 on ferroptosis susceptibility, we treated GC cells with different concentrations of Erastin, a ferroptosis inducer. The results showed that at equivalent Erastin concentrations, compared with the respective controls, GLUT3 KD significantly decreased the viability of AGS and HGC27 cells. In contrast, GLUT3 OE significantly increased cell viability in MKN45 cells** (Figure 3C)**. Furthermore, we observed that GLUT3 KD substantially elevated total cellular ROS levels in AGS and HGC27 cells, whereas GLUT3 OE significantly reduced total ROS levels in MKN45 cells. Strikingly, Erastin treatment further amplified these differences **(Figure 3D)**. Critically, in AGS-shGLUT3, HGC27-shGLUT3, and MKN45-Vector cells, the ferroptosis inhibitor ferrostatin-1 (Fer-1) significantly reduced the ROS levels. When co-administered with Erastin, Fer-1 effectively reversed ROS overproduction and blocked ferroptosis** (Figure S6A)**. This finding confirms that GLUT3 deficiency triggers ROS accumulation through the ferroptosis pathway.

Analysis of the cellular redox state revealed that, compared with controls, GLUT3 KD significantly increased lipid peroxidation levels and malondialdehyde (MDA) content while decreasing the reduced glutathione/oxidized glutathione (GSH/GSSG) ratio. Conversely, GLUT3 OE significantly decreased lipid peroxidation and the MDA content while increasing the GSH/GSSG ratio** (Figure 3E-G)**. Transmission electron microscopy (TEM) revealed that compared with HGC27-shNC control cells without Erastin treatment, HGC27-shGLUT3 cells exhibited a more typical ferroptotic morphology, including mitochondrial shrinkage and a reduction or disappearance of mitochondrial cristae. Upon Erastin exposure, these ferroptotic features became markedly more evident in HGC27-shGLUT3 cells **(Figure 3H)**. Collectively, these results demonstrate that GLUT3 plays a key role in regulating redox homeostasis and suppressing ferroptosis in GC cells.

Paclitaxel, a classical microtubule-stabilizing agent, exerts its antitumor effects primarily by disrupting mitosis and inducing apoptosis^14^. Building upon recent findings demonstrating that paclitaxel increases ROS levels and may contribute to ferroptosis induction^15^-^17^, we aimed to investigate whether GLUT3 mediates paclitaxel-induced ferroptosis and its effect on chemosensitivity. Intriguingly, under paclitaxel treatment, GLUT3 KD significantly increased cell death in AGS and HGC27 cells. Conversely, GLUT3 OE reduced cell death in MKN45 cells **(Figure S7A-C)**. Given that elevated MDA levels and a decreased reduced GSH/GSSG ratio are established biomarkers of ferroptosis, we further assessed these markers. As expected, GLUT3 KD significantly potentiated the ferroptosis-inducing effect of paclitaxel, as evidenced by increased intracellular MDA levels and a decreased GSH/GSSG ratio. In contrast, GLUT3 OE significantly attenuated MDA accumulation and increased the GSH/GSSG ratio upon paclitaxel treatment **(Figure S7D-I)**. Collectively, these results indicate that GLUT3 reduces the sensitivity of GC cells to paclitaxel by suppressing ferroptosis, suggesting that GLUT3 contributes to the development of chemoresistance.

### 3.5 The GLUT3-HK3-G6P axis drives glycolytic flux and lactate accumulation to suppress ferroptosis

**Figure 4:**
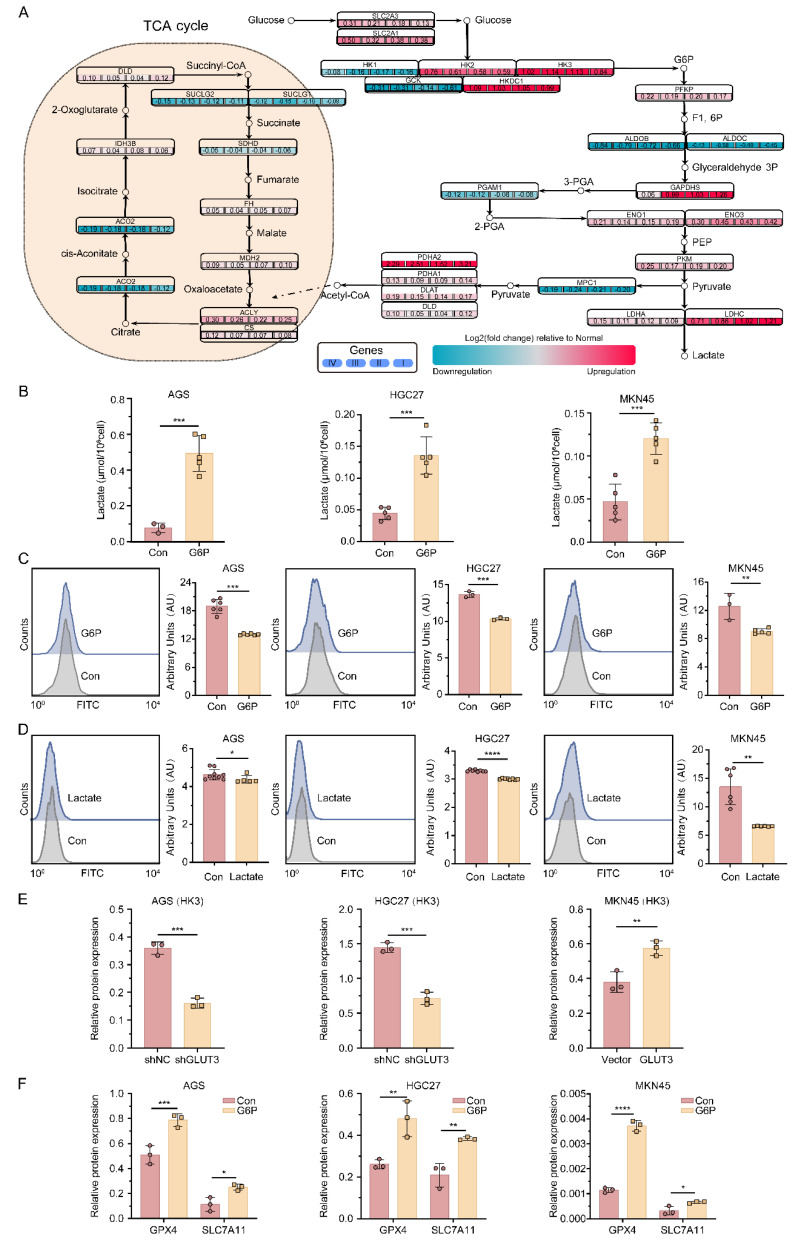
** The GLUT3-HK3-G6P-lactate axis reprograms glucose metabolism, driving ferroptosis resistance.** A) Integrated metabolic network map of key pathways and GLUT3 regulation that integrates genes and metabolites associated with the TCA cycle, glycolysis/gluconeogenesis pathways, and downstream regulation of GLUT3. B) Wild-type gastric cancer cells (AGS, HGC27, MKN45) were treated with 500 μM G6P for 24 h. Intracellular lactate was quantified with a Lactate Assay Kit. C) Lipid ROS levels were determined by flow cytometry in G6P-stimulated (500 μM, 24 h) wild-type gastric cancer cells. Representative histograms (left) and quantification (right) are shown. D) Lipid ROS levels were determined by flow cytometry in lactate-stimulated (15 mM, 24 h) wild-type gastric cancer cells. Representative histograms (left) and quantification (right) are shown. E) HK3 protein expression was quantified by Western blotting. F) GPX4 and SLC7A11 protein expression was quantified with or without G6P stimulation (500 μM) for 24 h by Western blotting. All the data are presented as the mean ± SD. *P<0.05, **P<0.01, ***P<0.001, ****P<0.0001. Abbreviations: ROS: Reactive Oxygen Species; G6P: Glucose-6-phosphate.

By integrating gene expression profiles from the TCGA-STAD cohort with metabolite information from a gastric cancer metabolomics dataset^18^, we detected significant increases in the mRNA levels of the key glycolysis genes HK3 and LDHC across stages I-IV in patients with GC **(Figure 4A)**. This change was accompanied by accumulation of the corresponding metabolites, G6P and lactate. Notably, although LDHC transcript levels were elevated, LDHC protein expression did not significantly differ between the GLUT3-KD or GLUT3-OE groups and their respective controls **(Figure S8A)**. In contrast, HK3 protein expression decreased in GLUT3-KD AGS and HGC27 cells but increased in GLUT3-OE MKN45 cells **(Figure 4E, S8B)**. Furthermore, stimulation with exogenous G6P for 24 h significantly increased intracellular lactate levels in AGS, HGC27, and MKN45 cells** (Figure 4B)**. These results indicate that GLUT3 specifically activates HK3 to drive glycolytic metabolic reprogramming, promoting the accumulation of the key intermediate metabolite G6P and its end product, lactate.

To validate the functional role of the core metabolites G6P and lactate in ferroptosis in GC cells, we performed functional assays in the AGS, HGC27, and MKN45 cell lines. A 24-h stimulation with exogenous G6P not only significantly upregulated the expression of GPX4 and SLC7A11 **(Figure 4F, S8C)** but also significantly reduced lipid ROS levels **(Figure 4C)**. Additionally, stimulation with exogenous lactate similarly effectively suppressed intracellular lipid ROS accumulation **(Figure 4D)**. In summary, metabolic reprogramming driven by the GLUT3-HK3-G6P axis leads to significant lactate accumulation in GC cells. Collectively, our data demonstrate that lactate accumulation functions as a ferroptosis suppressor by scavenging lipid ROS through redox homeostasis. Mechanistically, this protection may involve lactate-driven posttranslational modifications that modulate ferroptosis sensitivity.

### 3.6 Lactate transcriptionally activates MAPKAP1 via p300-mediated H3K18la to suppress ferroptosis

**Figure 5:**
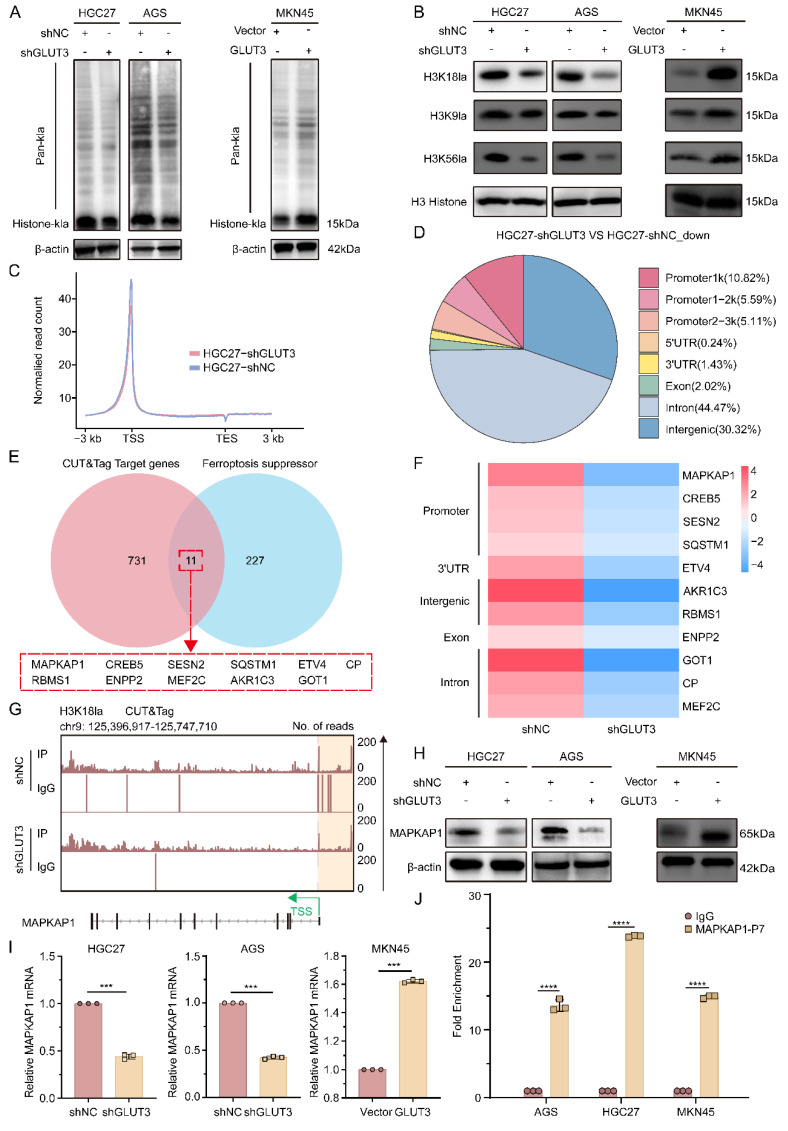
** GLUT3 directly activates MAPKAP1 transcription by facilitating H3K18la-specific binding at its promoter.** A) Pan-Kla protein expression in AGS/shGLUT3, HGC27/shGLUT3, MKN45/GLUT3 cells and their negative control cells. B) Protein expression of H3K18la, H3K9la, and H3K56la in AGS/shGLUT3, HGC27/shGLUT3, MKN45/GLUT3 cells and their corresponding negative control cells. C) Normalized read counts of shGLUT3 and shNC binding peaks in CUT&Tag sequencing data. D) Pie chart showing the genomic distribution of GLUT3 knockdown-associated downregulated peaks. E) Venn diagram depicting the overlap (n=11 genes, *P<0.05*) between H3K18la-specific CUT&Tag target genes downregulated in HGC27-shGLUT3 cells compared with HGC27-shNC control cells and a defined set of known ferroptosis suppressor genes. F) Heatmap of ferroptosis-related gene changes in the CUT&Tag data. G) IGV tracks from CUT&Tag analysis showing H3K18la enrichment at the promoter of MAPKAP1. H) Protein expression of MAPKAP1 in the indicated gastric cancer cells. I) The mRNA expression of MAPKAP1 in the indicated gastric cancer cells. J) ChIP‒qPCR assay of H3K18la status in MAPKAP1 promoter region 7 in AGS, HGC27 and MKN45 cells. All the data are presented as the mean ± SD. **P< 0.01, ***P<0.001, ****P<0.0001.

To determine whether GLUT3 regulates histone lactylation, we assessed Pan-Kla levels in GLUT3-KD (AGS, HGC27) and GLUT3-OE (MKN45) cells. GLUT3 KD significantly reduced total lactylation, whereas GLUT3 OE markedly increased total lactylation **(Figure 5A)**. Further analysis of key histone lactylation sites (H3K18la, H3K9la, and H3K56la) revealed that H3K18la was the most significantly upregulated modification in the GLUT3-OE group **(Figure 5B)**, identifying it as the core modification site. Mechanistically, GLUT3 modulates H3K18la expression via the histone acetyltransferase p300. GLUT3 KD decreased p300 protein expression, whereas GLUT3 OE increased it **(Figure S10A-B)**. Furthermore, p300 knockdown in wild-type cells significantly suppressed H3K18la levels** (Figure S10C-D)**.

To identify H3K18la downstream targets, we performed H3K18la-specific CUT&Tag sequencing in HGC27-shNC and HGC27-shGLUT3 cells. As shown in **Figure 5C**, H3K18la enrichment was predominantly localized to promoter regions. Comparative analysis revealed that 21.52% of the downregulated H3K18la peaks in GLUT3-KD cells were annotated to promoters relative to those in controls **(Figure 5D)**. This profiling revealed downregulated target genes in shGLUT3 cells, 11 of which overlapped with targets of defined ferroptosis suppressors **(Figure 5E)**. Among these, the MAPKAP1 promoter exhibited the most pronounced reduction in the H3K18la signal upon GLUT3 KD **(Figure 5F)**. H3K18la CUT&Tag analysis confirmed robust enrichment of this histone lactylation marker at the MAPKAP1 promoter **(Figure 5G)**, with significantly reduced enrichment in shGLUT3 cells compared with shNC controls. These results indicate that GLUT3 directly regulates MAPKAP1 transcription via H3K18la.

Notably, GLUT3 KD significantly reduced both MAPKAP1 protein and mRNA expression, whereas GLUT3 OE significantly increased them **(Figure 5H-I, S10E)**. ChIP‒qPCR analysis across seven regions of the MAPKAP1 promoter confirmed specific enrichment of H3K18la at region 7 **(Figure 5J, S9A)**. Critically, this enrichment was blocked by treatment with the glycolysis inhibitors 2-deoxy-D-glucose (2-DG), oxamate (OX), and dichloroacetate (DCA)** (Figure S9B)**. Collectively, these findings reveal a novel mechanism whereby lactate, via p300-mediated H3K18 lactylation, directly transcriptionally activates the key ferroptosis resistance factor MAPKAP1. This study is the first to elucidate how metabolic reprogramming drives epigenetic remodeling, specifically through histone lactylation, to increase ferroptosis resistance in GC cells.

### 3.7 GLUT3 drives GC cell growth and antagonizes paclitaxel-induced ferroptosis by regulating MAPKAP1

**Figure 6:**
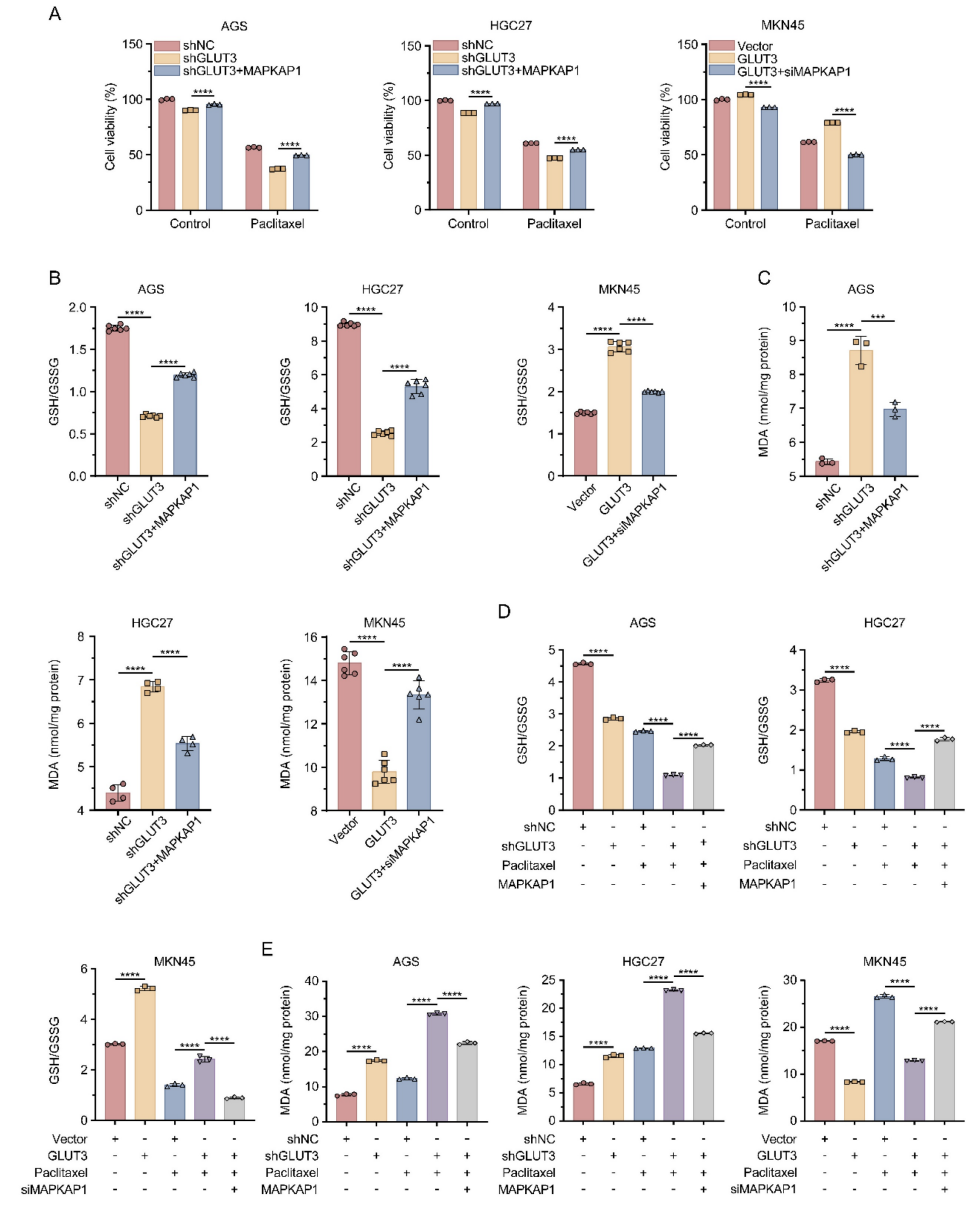
**GLUT3 inhibits paclitaxel-induced ferroptosis by activating MAPKAP1 in GC cells.** A) GLUT3-knockdown/overexpression GC cells transfected with or without MAPKAP1 plasmids/MAPKAP1-siRNA were treated or not treated with paclitaxel (AGS/HGC27: 0.1 μM; MKN45: 10 μM) for 24 h, after which cell viability was determined with a CCK8 assay. B) The ratio of GSH/GSSG in GLUT3-knockdown/overexpression GC cells transfected with or without MAPKAP1 plasmids/MAPKAP1-siRNA was determined by a microplate spectrophotometer. C) The levels of MDA in GLUT3-knockdown/overexpression GC cells transfected with or without MAPKAP1 plasmids/MAPKAP1-siRNA were determined with a microplate spectrophotometer. D) Control and GLUT3-knockdown GC cells were transfected with or without MAPKAP1 plasmids and treated with DMSO or paclitaxel (0.1 μM) for 24 h, and the ratio of GSH/GSSG was determined with a microplate spectrophotometer. Vector- and GLUT3-overexpressing cells were transfected with or without MAPKAP1 siRNA and treated with DMSO or paclitaxel (10 μM) for 24 h, and the ratio of GSH/GSSG was determined by a microplate spectrophotometer. E) Control and GLUT3-knockdown GC cells were transfected with or without MAPKAP1 plasmids and treated with DMSO or paclitaxel (0.1 μM) for 24 h, and the levels of MDA were determined with a microplate spectrophotometer. Vector- and GLUT3-overexpressing cells were transfected with or without MAPKAP1 siRNA and treated with DMSO or paclitaxel (10 μM) for 24 h, after which the levels of MDA were determined with a microplate spectrophotometer. All the data are presented as the mean ± SD. ***P< 0.001, ****P<0.0001. Abbreviations: MDA: Malondialdehyde; GSH: Glutathione (reduced); GSSG: Glutathione Disulfide (oxidized); CCK8: Cell Counting Kit-8.

To investigate the functional role of MAPKAP1 in GLUT3-mediated GC cell growth and ferroptosis resistance, we altered MAPKAP1 expression by transfecting GLUT3-OE cells with MAPKAP1-siRNA and GLUT3-KD cells with the MAPKAP1-OE plasmid. The knockdown and overexpression efficiency of MAPKAP1 was validated by Western blotting **(Figure S10F)**. The results revealed that MAPKAP1 OE significantly increased the GSH/GSSG ratio and suppressed the increase in MDA levels in GLUT3-KD GC cells. Conversely, suppression of MAPKAP1 decreased the GSH/GSSG ratio in GLUT3-OE cells and reversed the reduction in MDA levels **(Figure 6B-C)**. To determine whether GLUT3 modulates paclitaxel-induced ferroptosis through MAPKAP1, we repeated these experiments under paclitaxel treatment. MAPKAP1 OE rescued the growth inhibition induced by paclitaxel in GLUT3-KD cells. Conversely, suppression of MAPKAP1 expression sensitized GLUT3-OE cells to paclitaxel-induced growth suppression **(Figure 6A)**. Consistent with these findings, in response to paclitaxel treatment, GLUT3 KD significantly increased MDA levels and decreased the GSH/GSSG ratio in AGS and HGC27 cells compared with those in the controls; these effects were reversed by MAPKAP1 OE. In contrast, GLUT3 OE effectively prevented the increase in MDA levels and increased the GSH/GSSG ratio in paclitaxel-treated MKN45 cells, and this protective effect was abrogated by the suppression of MAPKAP1 **(Figure 6D-E)**. Collectively, these results establish that GLUT3 promotes GC cell growth and antagonizes paclitaxel-induced ferroptosis in a MAPKAP1-dependent manner.

### 3.8 GLUT3 inhibition sensitizes cells to paclitaxel for GCPM therapy by promoting ferroptosis

**Figure 7:**
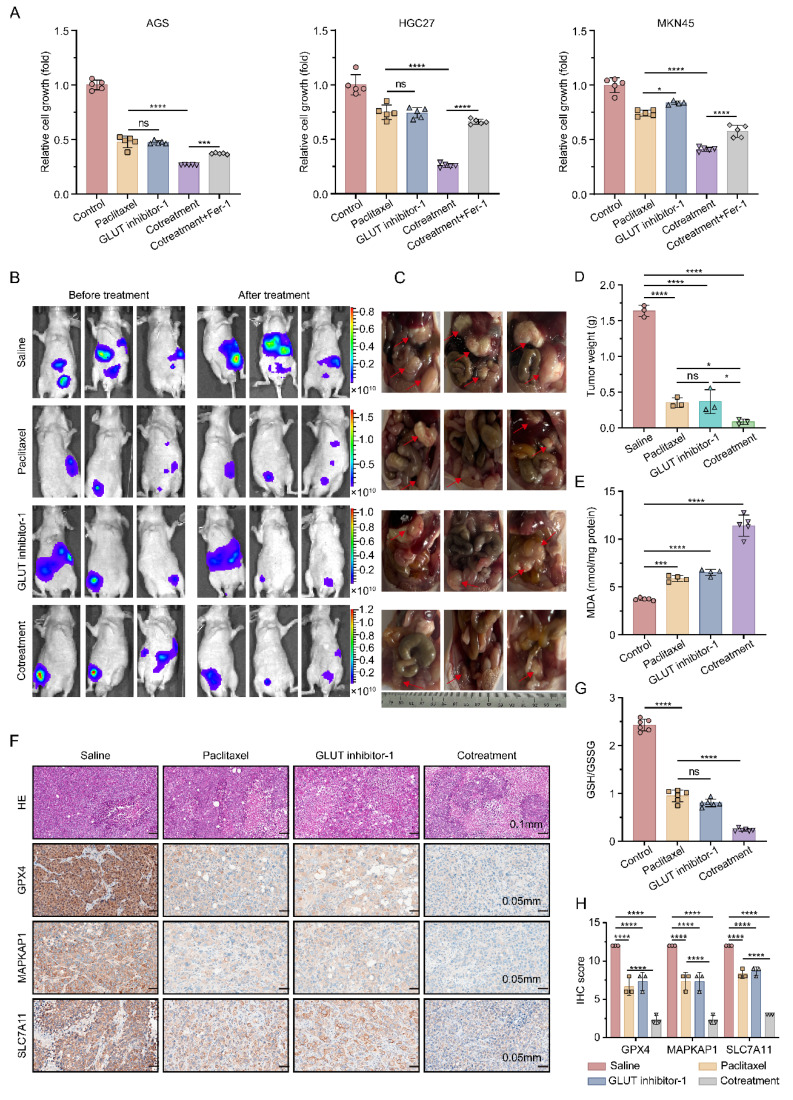
** GLUT3 inhibition sensitizes GC cells to paclitaxel in the context of peritoneal metastasis by promoting ferroptosis.** A) GC cells were treated with saline, paclitaxel (AGS/HGC27: 0.1 μM; MKN45: 10 μM), a GLUT inhibitor-1 (1 μM), a combination of GLUT inhibitor-1 and paclitaxel and a combination of GLUT inhibitor-1, paclitaxel and Fer-1 (2 μM) for 24 h. Subsequently, cell viability was measured with a CCK8 assay. B) Representative *in vivo* bioluminescence images of nude mice before and after treatment. Mice were intraperitoneally injected with MKN45-GLUT3 cells (1×10⁷ cells in 200 μL of PBS). The pretreatment images were captured at 21 days post-injection. Posttreatment images were taken on day 35 (24 h after the last treatment) with the indicated agents: saline, paclitaxel, GLUT inhibitor-1, and a combination of GLUT inhibitor-1 and paclitaxel. C) Gross morphology of peritoneal metastatic nodules at the experimental endpoint (day 35): representative specimens from the saline, paclitaxel (15 mg/kg), GLUT inhibitor-1 (10 mg/kg), and combination therapy groups. The red arrows indicate gastric cancer peritoneal metastases. D) Weight quantification of peritoneal metastatic tumors. E) The levels of MDA in tumor tissues from the indicated groups were determined. F) Representative H&E staining and IHC staining images of GPX4, SLC7A11, and MAPKAP1 in tumor tissues from the indicated groups are shown. Scale bar: 100 μm and 50 μm. G) The ratio of GSH/GSSG in tumor tissues from the indicated groups was determined. H) Quantification of the protein expression of GPX4, SLC7A11, and MAPKAP1 in tumor tissues from the indicated groups (n=3). All the data are presented as the mean ± SD. *P<0.05, ***P<0.001, ****P<0.0001, ns: not significant. Immunohistochemical staining was quantified using the H-score, calculated as staining intensity (0-3) × percentage of positive cells (1-4). The staining intensity was graded as follows: 0 (negative), 1 (weak), 2 (intermediate), or 3 (strong). The proportions of positively stained cells were categorized as follows: 1 (0-25%), 2 (26-50%), 3 (51-75%), or 4 (76-100%). Abbreviations: H&E: Hematoxylin and Eosin staining; IHC: Immunohistochemical; CCK8: Cell Counting Kit-8; Fer-1: Ferrostatin-1; MDA: Malondialdehyde; GSH: Glutathione (reduced); GSSG: Glutathione Disulfide (oxidized).

Building upon the critical role of GLUT3 in mediating ferroptosis resistance in gastric cancer, we further evaluated the therapeutic potential of the GLUT3 inhibitor GLUT inhibitor-1 in promoting ferroptosis and sensitizing paclitaxel treatment for GCPM. *In vitro* experiments demonstrated that compared with paclitaxel monotherapy, combining GLUT inhibitor-1 with paclitaxel had a significantly stronger inhibitory effect on cell viability in AGS, HGC27, and MKN45 cells. This synergistic effect was reversed by cotreatment with Fer-1, indicating its dependence on ferroptosis pathway activation** (Figure 7A)**. To validate the *in vivo* efficacy, we used a nude mouse GCPM model established with GLUT3-OE MKN45 cells. Treatments (paclitaxel monotherapy, GLUT inhibitor-1 monotherapy, or combination therapy) were initiated on day 22 post-inoculation and administered every other day (qod) for a total of 7 doses, with the final dose given on day 34. The mice were euthanized on day 35 post-inoculation (24 h after the last administration) for final tumor assessment. At this endpoint, compared with either monotherapy group, the combination treatment group exhibited significantly greater suppression of tumor growth **(Figure 7B-D)**. Biochemical analysis of tumor tissues revealed characteristic ferroptotic features in the combination group: elevated levels of the lipid peroxidation product MDA **(Figure 7E)**, a decreased reduced GSH/GSSG ratio **(Figure 7G)**, and significant downregulation of the ferroptosis resistance molecules MAPKAP1, SLC7A11, and GPX4 **(Figure 7F, 7H)**. These results collectively demonstrate that a GLUT inhibitor-1 increases the preclinical efficacy of paclitaxel against GCPM through the inhibition of GLUT3 to promote ferroptosis.

To validate the correlation between GLUT3 and MAPKAP1 in gastric cancer, we performed IHC staining analysis on paired tumor and adjacent normal tissues from GC patients using tissue microarrays. On the basis of predefined IHC scoring criteria, patients were stratified into four groups: Group I (low MAPKAP1/low GLUT3, n=56); Group II (high MAPKAP1/low GLUT3, n=11); Group III (low MAPKAP1/high GLUT3, n=79); and Group IV (high MAPKAP1/high GLUT3, n=31)** (Figure S11A)**. Kaplan-Meier survival analysis revealed that GC patients with high GLUT3 expression had a significantly shorter OS than patients with low GLUT3 expression did, regardless of MAPKAP1 expression status **(Figure S11B)**. Among the four stratified groups, Group IV (high MAPKAP1/high GLUT3) had the shortest OS **(Figure S11B)**. Collectively, these clinical data not only validate the coexpression pattern of GLUT3 and MAPKAP1 in gastric cancer but also confirm that high GLUT3 expression is a core determinant of poor prognosis in patients with GC, while co-overexpression of GLUT3 and MAPKAP1 synergistically exacerbates survival outcomes.

## 4. Discussion

Despite the widespread clinical application of paclitaxel-based chemotherapy in GCPM treatment, drug resistance remains a critical challenge. Ferroptosis, an iron-dependent form of cell death driven by lipid peroxidation, represents a promising strategy for overcoming therapeutic resistance because of its distinct mechanism. Comprehensive investigations of the regulatory mechanisms of ferroptosis, the identification of druggable targets, and the development of novel therapies are urgently needed to improve GCPM management in the clinic.

Our study demonstrated that GLUT3 expression was significantly upregulated in paclitaxel-resistant GCPM tissues. Elevated GLUT3 expression was correlated with poor prognosis in GC patients. Notably, we identified a previously unrecognized function of GLUT3: it suppressed ferroptosis by reducing intracellular lipid ROS levels, thereby promoting peritoneal metastasis progression and conferring paclitaxel resistance. Mechanistically, GLUT3 upregulated HK3 expression, promoting the accumulation of G6P and lactate. The accumulation of lactate supported p300-mediated H3K18la, which transcriptionally upregulated MAPKAP1 expression and ultimately inhibited ferroptosis in GC cells. Collectively, these findings suggest that GLUT3 drives the novel GLUT3-HK3-G6P-lactate-H3K18la-MAPKAP1 axis to suppress ferroptosis and promote peritoneal metastasis progression and chemoresistance. Therefore, targeting GLUT3 to induce ferroptosis may represent a novel therapeutic strategy to inhibit peritoneal metastasis growth and restore paclitaxel sensitivity.

Previous studies have established that GLUT3 is a high-affinity glucose transporter that is overexpressed in gastric cancer, where it increases glycolytic flux and is associated with poor prognosis^19^. Unlike the ubiquitously expressed GLUT1, GLUT3 maintains efficient glucose uptake under nutrient deprivation conditions, promoting tumor cell survival^20^. Analysis of 150 gastric cancer specimens revealed the expression of GLUT1, GLUT3, GLUT6, and GLUT10 in 22.0%, 66.0%, 38.0%, and 43.3% of cases, respectively. Among these four glucose transporters, GLUT3 exhibited the highest positivity rate and the strongest association with adverse outcomes^21^. GLUT3 also upregulates lactate dehydrogenase A (LDHA) to increase lactate production, catalyzing H3K18la, which activates epithelial-mesenchymal transition (EMT) pathways and increases metastatic capacity^19^. Furthermore, H3K18la upregulates vascular cell adhesion molecule 1 (VCAM1) transcription, activating the AKT-mTOR signaling pathway to promote gastric cancer cell proliferation, EMT, and metastasis^22^. Therefore, deciphering the downstream effectors of GLUT3, which orchestrates not only glucose metabolism but also epigenetic modifications and pro-oncogenic signaling pathways, is crucial for understanding the multifaceted drivers of gastric cancer malignancy.

The central role of GLUT3 in tumor metabolism is well documented. In ovarian cancer, zinc finger E-box binding homeobox 1 (ZEB1) binding activates GLUT3 transcription to promote glycolysis and metastasis^23^. Studies on pancreatic cancer have shown that sirtuin 7 (SIRT7) regulates H3K122 succinylation at GLUT3 enhancer regions, affecting chemosensitivity^24^. Research on colorectal cancer has demonstrated that caveolin 1 (CAV1) overexpression increases high mobility group AT-hook 1 (HMGA1)-mediated GLUT3 transcription to drive aerobic glycolysis^25^. Studies on pancreatic ductal adenocarcinoma have revealed that tumor-associated macrophages promote glycolysis through the IL-8/STAT3/GLUT3 signaling pathway^26^. While existing research focuses primarily on the regulation of GLUT3 expression and its core glycolytic functions, how GLUT3-driven metabolic flux regulates ferroptosis resistance remains unclear. Our study provides the first evidence that GLUT3 drives HK3 expression, establishing upstream validation for the GLUT3-HK3-LDHA pathway and revealing a crucial novel biological function in which GLUT3 regulates ferroptosis through HK3-mediated glycolysis enhancement and lactate accumulation.

Building upon the key finding of GLUT3-driven lactate accumulation, we further investigated downstream effects of lactate. Lactate drives protein lactylation modification: lactoyl-CoA synthesized by acyl-CoA synthetase short chain family member 2 (ACSS2) is transferred by p300 to lysine residues on target proteins, directly regulating gene transcription^27^-^29^. Studies report that lactate promotes p300-mediated H3K18la binding to the methyltransferase-like 3 (METTL3) promoter, modulating N6-methyladenosine (m6A) modification levels. METTL3-mediated m6A modification increases the expression of acyl-CoA synthetase long chain family member 4 (ACSL4) messenger RNA, increasing its stability through YTH domain containing 1 (YTHDC1)-dependent mechanisms to upregulate ACSL4 and promote ferroptosis^28^. H3K18la increases the transcriptional activity of NFS1 cysteine desulfurase (NFS1), suppressing ferroptosis in liver cancer cells^30^. Acetyl-CoA acetyltransferase 2 (ACAT2)-mediated lactylation modification of the glutamate-cysteine ligase modifier subunit (GCLM) promotes ferroptosis resistance in KRASG12D-mutant tumors^31^. These studies reveal the molecular mechanisms of lactylation modification in ferroptosis regulation. In our study, lactate accumulated through the GLUT3-HK3 axis induced the H3K18la modification via p300. As an activating histone modification, H3K18la likely upregulates MAPKAP1 transcription by increasing chromatin accessibility or recruiting transcription factors, thereby inhibiting ferroptosis in gastric cancer cells. This work establishes the role of the lactate-H3K18la-MAPKAP1 axis in ferroptosis regulation and paclitaxel resistance, addressing knowledge gaps in metabolic‒epigenetic crosstalk governing cancer drug resistance. Future studies should investigate the precise mechanisms through which MAPKAP1 inhibits ferroptosis.

How does MAPKAP1 inhibit ferroptosis? MAPKAP1 serves as an indispensable core scaffold protein for mammalian target of rapamycin complex 2 (mTORC2), which is essential for maintaining its structural integrity and function^32^-^34^. The core function of mTORC2 involves phosphorylating AKT at Ser473 to achieve full activation. Notably, activated AKT reciprocally phosphorylates the Thr86 residue of MAPKAP1, increasing mTORC2 kinase activity and establishing a positive feedback loop that causes persistent AKT pathway activation^35^,^36^. Research has confirmed that activated AKT signaling plays a central role in conferring ferroptosis resistance: activated AKT regulates glycogen synthase kinase 3 beta (GSK3B) to influence nuclear factor erythroid 2-related factor 2 (NRF2) activity; NRF2 then suppresses ferroptosis by regulating key antioxidant genes, including HO-1, GPX4, and SLC7A11^37^-^39^. Therefore, H3K18la-mediated MAPKAP1 upregulation likely constitutes an important upstream mechanism through which ferroptosis is inhibited through the MAPKAP1-mTORC2-AKT-NRF2 axis. Current evidence linking MAPKAP1 to ferroptosis regulation primarily depends on its role in maintaining mTORC2 complex function. Reports indicate that mitogen-activated protein kinase 14 (MAPK14) phosphorylates MAPKAP1 to promote mTORC2 assembly, activating AKT to inhibit ferroptosis^40^. Although MAPKAP1 is overexpressed in multiple cancers and drives malignant progression^41^-^44^, whether it directly interacts with ferroptosis execution proteins such as GPX4, ACSL4, or FSP1 and regulates their activity independently of the mTORC2-AKT pathway remains unknown. Future research should analyze the molecular mechanisms underlying direct ferroptosis regulation by MAPKAP1 to elucidate its noncanonical functions, providing a foundation for the development of precision therapies targeting specific domains.

In summary, our study reveals a novel pathway through which GLUT3 drives GCPM progression and paclitaxel resistance: the GLUT3-HK3-G6P-lactate-H3K18la-MAPKAP1 signaling axis suppresses ferroptosis. Targeting key nodes of this axis, including developing high-selectivity GLUT3 inhibitors, exploring strategies to specifically disrupt H3K18la modification by blocking p300 catalytic activity or developing inhibitors recognizing this modification, and establishing precision therapies targeting the mTORC2 scaffold protein MAPKAP1, may induce ferroptosis, sensitize tumors to paclitaxel, and overcome peritoneal metastasis. Despite the challenges associated with the physiological functions of GLUT3 and the technical difficulties associated with targeting histone lactylation, this newly identified axis represents a promising therapeutic direction against gastric cancer peritoneal dissemination and chemotherapy resistance.

## Supplementary Material

Supplementary figures and tables.

## Data Availability

The data supporting the conclusions of this study are included in the supplementary material files and available from the corresponding author on request.

## Abbreviations

GLUT3: Glucose transporter type 3
H3K18la: Histone H3 lysine 18 lactylation
MAPKAP1: Mitogen-activated protein kinase associated protein
GCPM: Gastric cancer peritoneal metastasis
GC: Gastric cancer
IPC: Intraperitoneal chemotherapy
GPX4: Glutathione peroxidase 4
SLC7A11: Solute carrier family 7 member 11
MDA: Malondialdehyde
GSH: Glutathione (reduced form)
GSSG: Glutathione disulfide (oxidized form)
ROS: Reactive oxygen species
HK3: Hexokinase 3
G6P: Glucose-6-phosphate
TEM: Transmission electron microscopy
p300: E1A binding protein p300 (EP300)
EMT: Epithelial-mesenchymal transition
mTORC2: Mammalian target of rapamycin complex 2
NRF2: Nuclear factor erythroid 2-related factor 2
NIPS: Combined intraperitoneal and intravenous paclitaxel plus S-1 regimen
FFPE: Formalin-fixed paraffin-embedded
TMA: Tissue microarray
shRNA: Short hairpin RNA
siRNA: Small interfering RNA
Fer-1: Ferrostatin-1
