# Supplementary material for: GLUT3 drives paclitaxel resistance in peritoneal metastatic gastric cancer by promoting H3K18 lactylation-mediated MAPKAP1 transcription to suppress ferroptosis
Source: Int J Biol Sci. 2026 Apr 23;22(9):4826–46. doi: 10.7150/ijbs.130059 (PMC13182558; doi:10.7150/ijbs.130059)
Supplement: Supplementary file 1 — Supplementary figures and tables. [file ijbsv22p4826s1.pdf]

Supplementary information for the manuscript

GLUT3 drives paclitaxel resistance in peritoneal metastatic gastric cancer by promoting H3K18 lactylation-mediated MAPKAP1 transcription to suppress ferroptosis

Ying Sun, Xirui Duan, Benyan Zhang, Cheng Xiong, Jun Ji, Qu Cai, Wang Yao, Jinling Jiang, Junwei Wu, Chao Wang, Liting Guo, Chenfei Zhou, Beiqin Yu, Feng Qi, Jun Zhang

✉ Corresponding authors: Jun Zhang, Email: junzhang10977@sjtu.edu.cn; Feng Qi, Email: qf12486@rjh.com.cn; Beiqin Yu, Email: ybq@sjtu.edu.cn

Supplementary Table

Table S1. Association between GLUT3 expression and clinicopathological characteristics.

Variable	Category	Gene		Total	X^2	P
		GLUT3-High	GLUT3-Low			
Age	≥65	62	31	93		
	<65	47	37	84	2.142	0.143
Total		109	68	177		
Gender	Male	66	41	107		
	Female	43	27	70	0.001	0.973
Total		109	68	177		
T stage	T1	1	4	5		
	T2	13	10	23		
	T3	64	45	109	8.566	0.036
	T4	31	9	40		
Total		109	68	177		
N stage	N0	14	29	43		
	N1	11	12	23		
	N2	27	12	39	27.525	<0.001
	N3	57	15	72		
Total		109	68	177		
M stage	M0	104	68	172		
	M1	5	0	5	3.21	0.073
Total		109	68	177		
Pathologic stage	Stage I-II	0	1	1		
	Stage II	22	18	40		
	Stage II-III	25	13	38	3.902	0.419
	Stage III	60	36	96		
	Stage III-IV	2	0	2		
Total		109	68	177		

Table S2. Multivariable Cox regression analysis adjusted for GLUT3^{High}/GLUT3^{Low} group, T stage, N stage and M stage.

	Variable	Univariate analysis		Multivariate analysis	
		HR (95% CI)	P-value	HR (95% CI)	P-value
Group	GLUT3^{LowR}<br>GLUT3^{High}	3.913(2.480-6.175)	< 0.001	2.717(1.689-4.372)	< 0.001
Age	< 65^{R}<br>≥65	0.935(0.649-1.348)	0.719		
Gender	Female^{R}<br>Male	0.801(0.552-1.160)	0.240		
T stage	T1+T2^{R}<br>T3+T4	4.024(1.959-8.267)	< 0.001	2.906(1.401-6.028)	0.004
N stage	N0+N1^{R}<br>N2+N3	5.082(3.168-8.150)	< 0.001	3.219(1.963-5.280)	< 0.001
M stage	M0^{R}<br>M1	4.854(1.931-12.202)	< 0.001	2.434(0.966-6.137)	0.059
Pathologic stage	I-II+II+II-III^{R}<br>III+III-IV	1.437(0.989-2.088)	0.057		

Annotation: HR: Hazard Ratio; R: Reference.

Table S3. Reagents used in the experiments.

Reagent	Reference number	Company
Erastin	HY15763	MedChemExpress
Ferrostatin-1	HY-100579	MedChemExpress
D-Glucose 6-phosphate disodium salt	HY-128374	MedChemExpress
Lactate	HY-B2227	MedChemExpress
2-DG	HY-13966	MedChemExpress
Oxamate	HY-W013032A	MedChemExpress
DCA	S8615	Selleck
Paclitaxel	HY-B0015	MedChemExpress
GLUT inhibitor-1	HY-139605	MedChemExpress

Table S4. siRNA sequences used in the experiments.

Gene	Sequence（5'-3'）
MAPKAP1(h)-siRNA-1	SS: GGGAAGCAGUCGAUAUUAU<br>AS: AUAAUAUCGACUGCUUCCC
MAPKAP1(h)-siRNA-2	SS: GAGUCACUCUUUGUUCGAA<br>AS: UUCGAACAAAGAGUGACUC
MAPKAP1(h)-siRNA-3	SS: GGGUCAUGUAGGUACAACA<br>AS: UGUUGUACCUACAUGACCC
EP300(h)-siRNA-1	SS: GAUGAAUUAAUCAACUCUA<br>AS: UAGAGUUGAUUAAUUCAUC
EP300(h)-siRNA-2	SS: CAUUUGCUAUGGACAAAAA<br>AS: UUUUUGUCCAUAGCAAAUG
EP300(h)-siRNA-3	SS: CUAGAGACACCUUGUAGUA<br>AS: UACUACAAGGUGUCUCUAG

Annotation: AS: Antisense; SS: Sense Strand.

Table S5. Antibodies used in western blot (WB) and immunohistochemistry (IHC) tests.

Antibody	Dilution rate	Reference number	Company
GLUT3	1:1000 (WB)	52310	Signalway Antibody
SLC7A11	1:1000 (WB)	T57046	Abmart
GPX4	1:1000 (WB)	T56959	Abmart
HK3	1:10000 (WB)	67803-1-Ig	Proteintech
LDHC	1:1000 (WB)	43519-1	Signalway Antibody
KAT3B/p300	1:1000 (WB)	AF5360	Affinity
L-Lactyl	1:1000 (WB)	PTM-1401RM	PTM BIO
H3K9la	1:1000 (WB)	PTM-1419RM	PTM BIO
H3K18la	1:1000 (WB)	PTM-1406RM	PTM BIO
H3K56la	1:1000 (WB)	PTM-1421RM	PTM BIO
Histone H3	1:2000 (WB)	4499S	Cell Signaling Technology
MAPKAP1	1:1000 (WB)	15463-1-AP	Proteintech
β-actin	1:20000 (WB)	66009-1-Ig	Proteintech
GLUT3	1:400 (IHC)	20403-1-AP	Proteintech
SLC7A11	1:200 (IHC)	26864-1-AP	Proteintech
GPX4	1:100 (IHC)	T56959	Abmart
MAPKAP1	1:250 (IHC)	15463-1-AP	Proteintech

Table S6. Primers used in this study.

Primer Name	Sequence (5'→3')
GLUT3-F	F: 5'-GCTGGGCATCGTTGTTGGA-3'
GLUT3-R	R: 5'-GCACTTTGTAGGATAGCAGGAAG-3'
MAPKAP1-F	F: 5'-GGTGGACACCGATTTCCCC-3'
MAPKAP1-R	R: 5'-CGCTTCACTGCCTTCAGTAAGA-3'
MAPKAP1（ChIP-DNA-F1）	F: 5'-CGTGCCCGGCCTATATAGAGAT-3'
MAPKAP1（ChIP-DNA-R1）	R: 5'-CAAAGCAGCAGGTGTGAACAAG-3'
MAPKAP1（ChIP-DNA-F2）	F: 5'-ACAGATAACCTGGTGACCTCAG-3'
MAPKAP1（ChIP-DNA-R2）	R: 5'-ACTGTAGGCAATTCTAACACAATGG-3'
MAPKAP1（ChIP-DNA-F3）	F: 5'-ATGATGGTGGTCCCCTAAGAT-3'
MAPKAP1（ChIP-DNA-R3）	R: 5'-GCCACTGCACTCCAGCCTG-3'
MAPKAP1（ChIP-DNA-F4）	F: 5'- CCTCATCATTAAGTGATGGATGACC-3'
MAPKAP1（ChIP-DNA-R4）	R: 5'-GGCTCACACCTGTAATCCCAG-3'
MAPKAP1（ChIP-DNA-F5）	F: 5'-TCTCAAGTGATCTGCCCTCCT-3'
MAPKAP1（ChIP-DNA-R5）	R: 5'-ACAATAATCTTCCTCAAGCCCTG-3'
MAPKAP1（ChIP-DNA-F6）	F: 5'-CAGTCCTCCTACATCTCCTGGAA-3'
MAPKAP1（ChIP-DNA-R6）	R: 5'-TGTTAGGCTTCTCCATCGAATCT-3'
MAPKAP1（ChIP-DNA-F7）	F: 5'-CGAACTAAGGGCTTTTCTCCGT-3'
MAPKAP1（ChIP-DNA-R7）	R: 5'-CCGAGCAGCAGCCCTATTAC-3'
β-actin-F	F: 5'-CATGTACGTTGCTATCCAGGC-3'
β-actin-R	R: 5'-CTCCTTAATGTCACGCACGAT-3'

Supplementary Figures

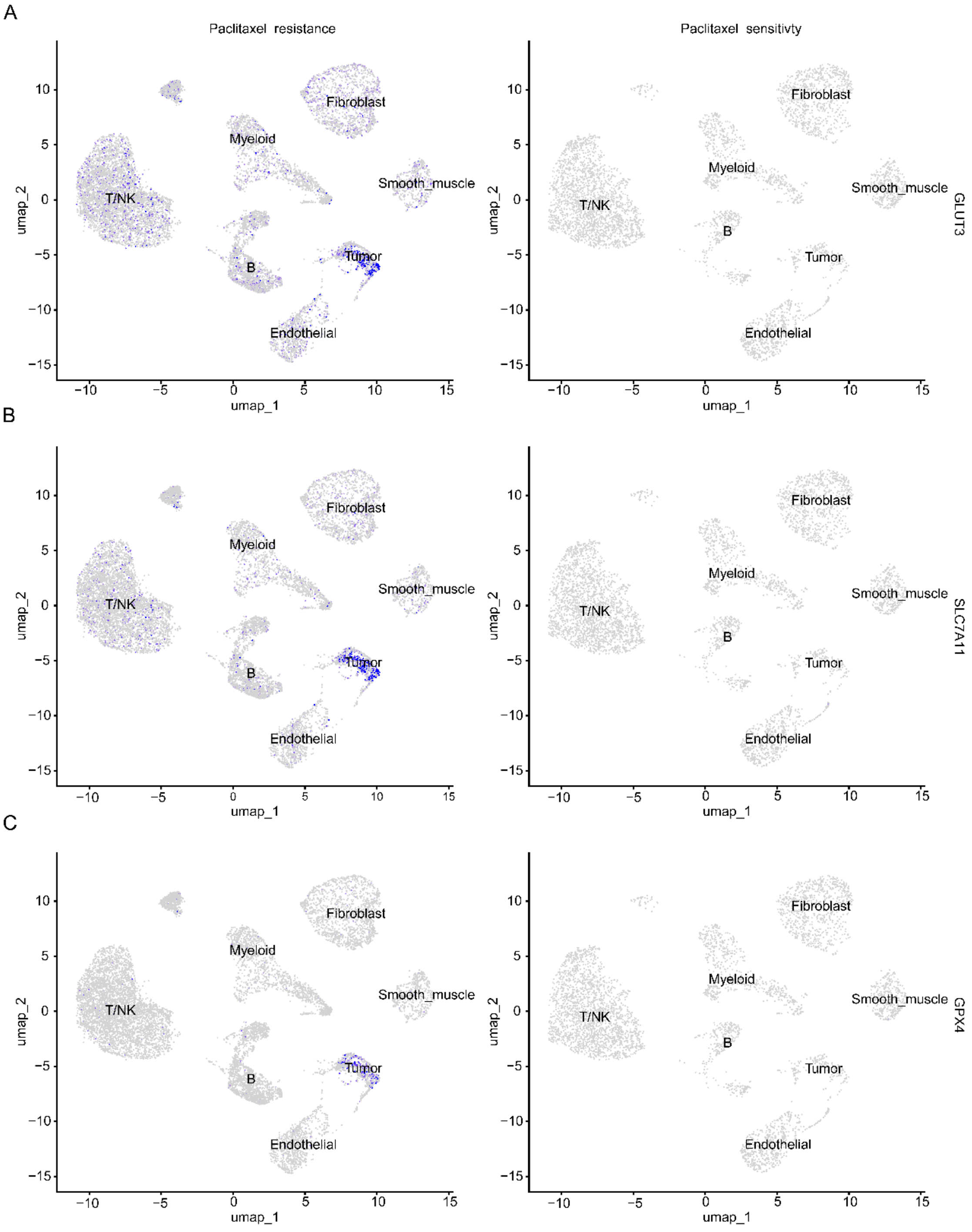

Figure S1. GLUT3 and the ferroptosis-resistance genes GPX4 and SLC7A11 are upregulated in paclitaxel-resistant GCPM tissues. A-C) UMAP plots showing the distribution and abundance of GLUT3, GPX4, and SLC7A11 in GCPM tissues with paclitaxel resistance or sensitivity.

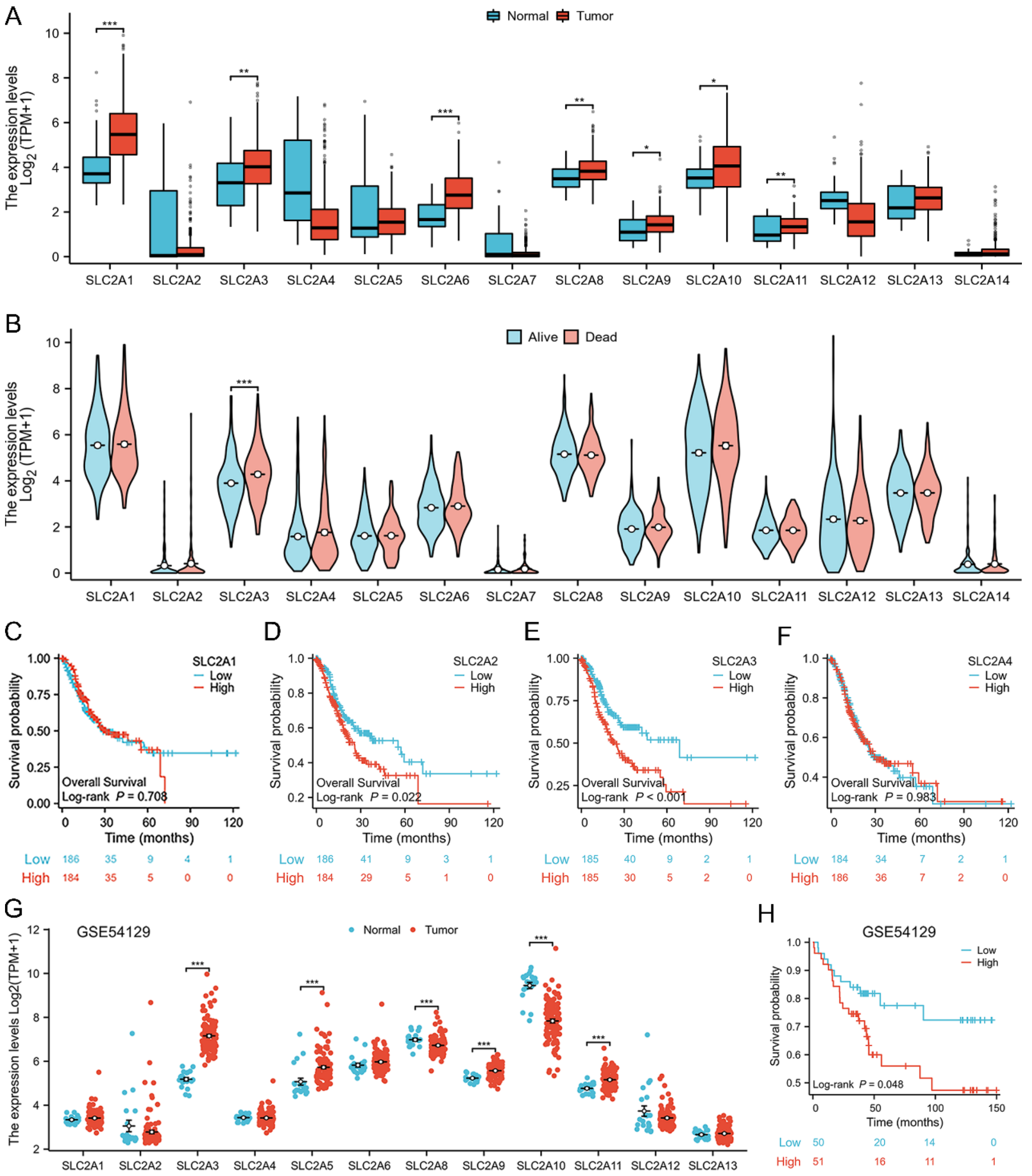

Figure S2. Comprehensive analysis of GLUT family gene expression and clinical significance in gastric cancer. A) Differential expression of GLUT1-GLUT14 in normal gastric tissues and gastric cancer tissues from TCGA cohort. B) Expression profiles of GLUT1-GLUT14 stratified by survival outcome (alive or dead) in TCGA cohort. C-F) Kaplan-Meier survival analysis for GLUT1-GLUT4 from TCGA cohort. G) Differential expression of glucose transporters (GLUT1-GLUT6, GLUT8-GLUT13) in normal gastric tissues and gastric cancer tissues from the GEO cohort. H) Kaplan-Meier survival analysis for GLUT3 from GEO cohort. All data are presented as the mean ± SD. *$P<0.05$*, **$P<0.01$*, ***$P<0.001$*.

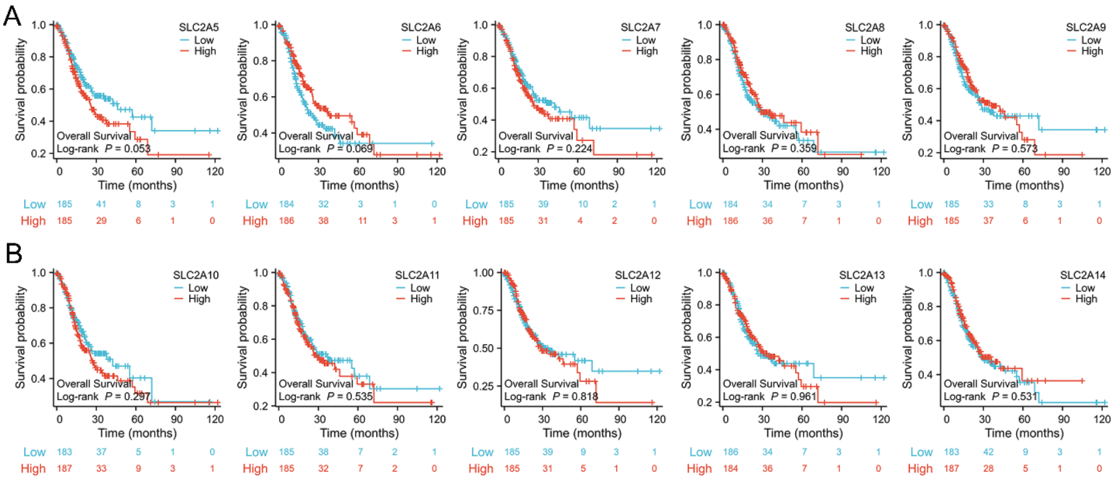

Figure S3. Kaplan-Meier survival curves for GLUT5 to GLUT14 in gastric cancer from TCGA cohort. A-B) Kaplan-Meier survival analysis for GLUT5-GLUT14 from TCGA cohort.

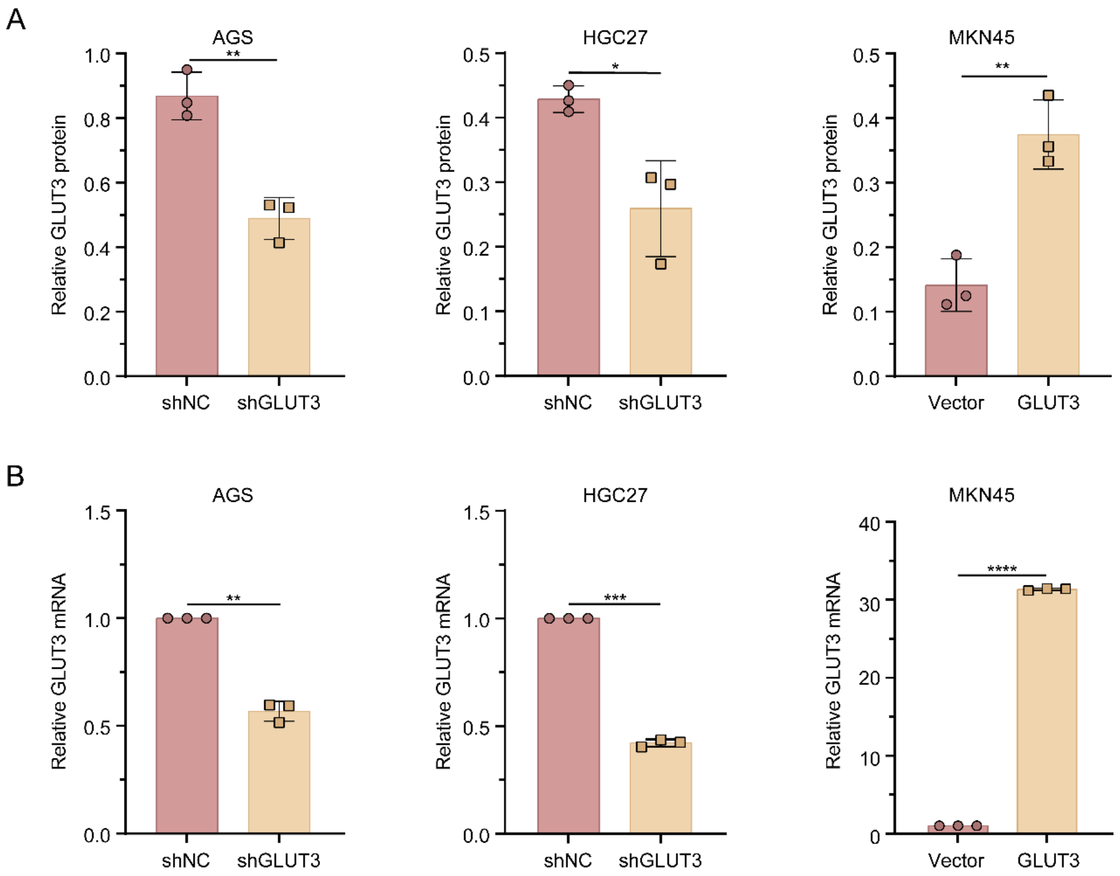

Figure S4. Validation of efficient GLUT3 knockdown (KD) and overexpression (OE). A-B) The knockdown and overexpression efficiencies of GLUT3 were validated at protein and mRNA levels in GC cells. All data are presented as the mean ± SD. **P< 0.05*, ***P<0.01*, ****P<0.001*, *****P<0.0001*.

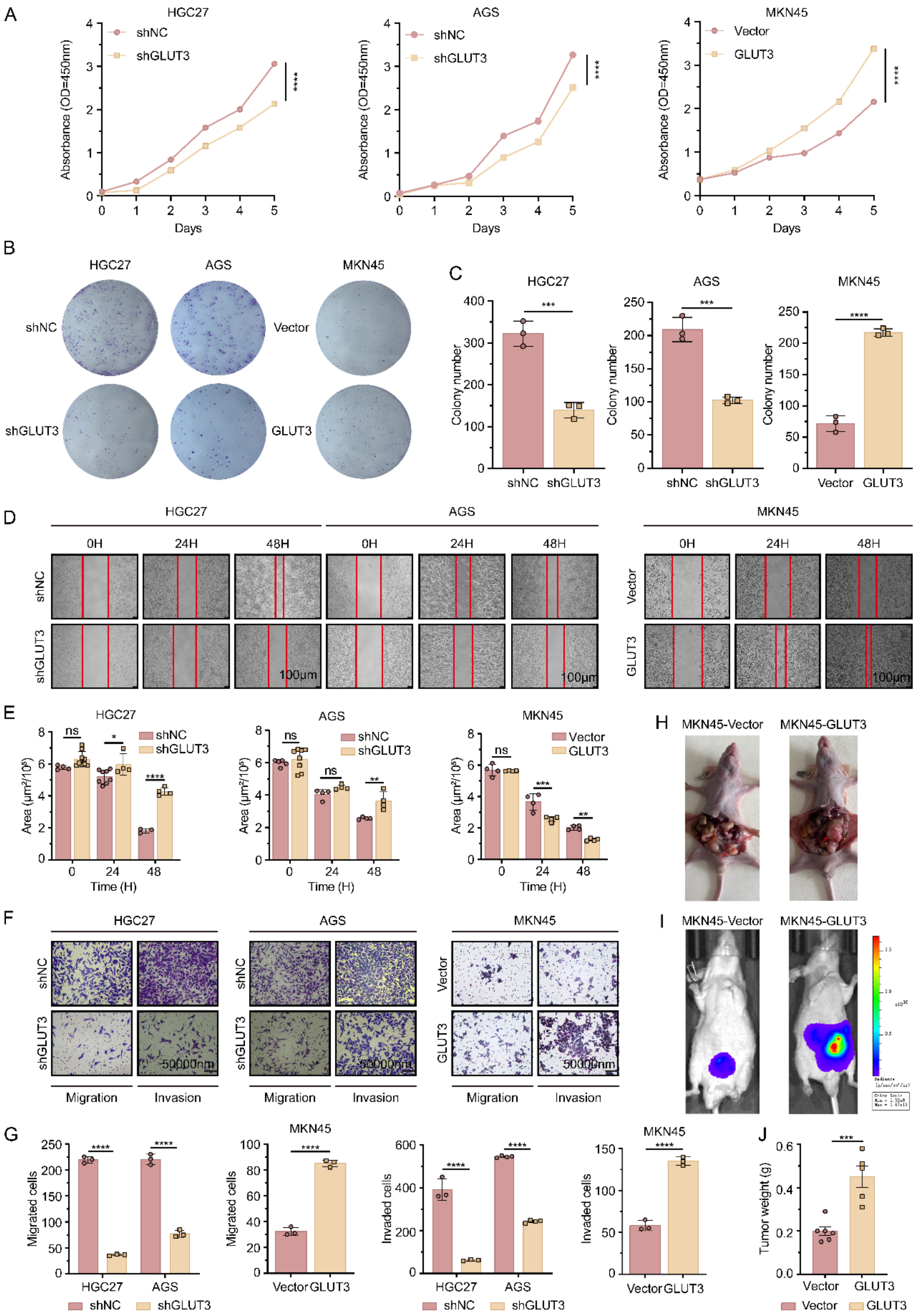

Figure S5. GLUT3 promotes GC growth and metastasis in vitro and in vivo. A) Effects of GLUT3 knockdown and overexpression on cell proliferation were determined by CCK8 assay. B-C) Effects of GLUT3 knockdown and overexpression

on cell proliferation were determined by colony formation assay. D-E) Wound healing assay showing the migration capacity of gastric cancer cells at 0 h, 24 h, and 48 h. F-G) Representative images and quantification of migrated and invaded gastric cancer cells through Transwell chambers. H) Representative in vivo macroscopic view images of nude mice at 21 days post-intraperitoneal injection of MKN45-Vector or MKN45-GLUT3 cells (1×10^7 cells in 200 μL PBS). The red arrows indicate gastric cancer peritoneal metastases. I) Representative in vivo bioluminescence images of nude mice at 21 days post-intraperitoneal injection of MKN45-Vector or MKN45-GLUT3 cells (1×10^7 cells in 200 μL PBS). J) Weight of peritoneal tumors harvested at Day 21. All data are presented as the mean ± SD. *$P<0.05$, **$P<0.01$, ***$P<0.001$, ****$P<0.0001$, ns: not significant. Abbreviations: CCK8: Cell Counting Kit-8.

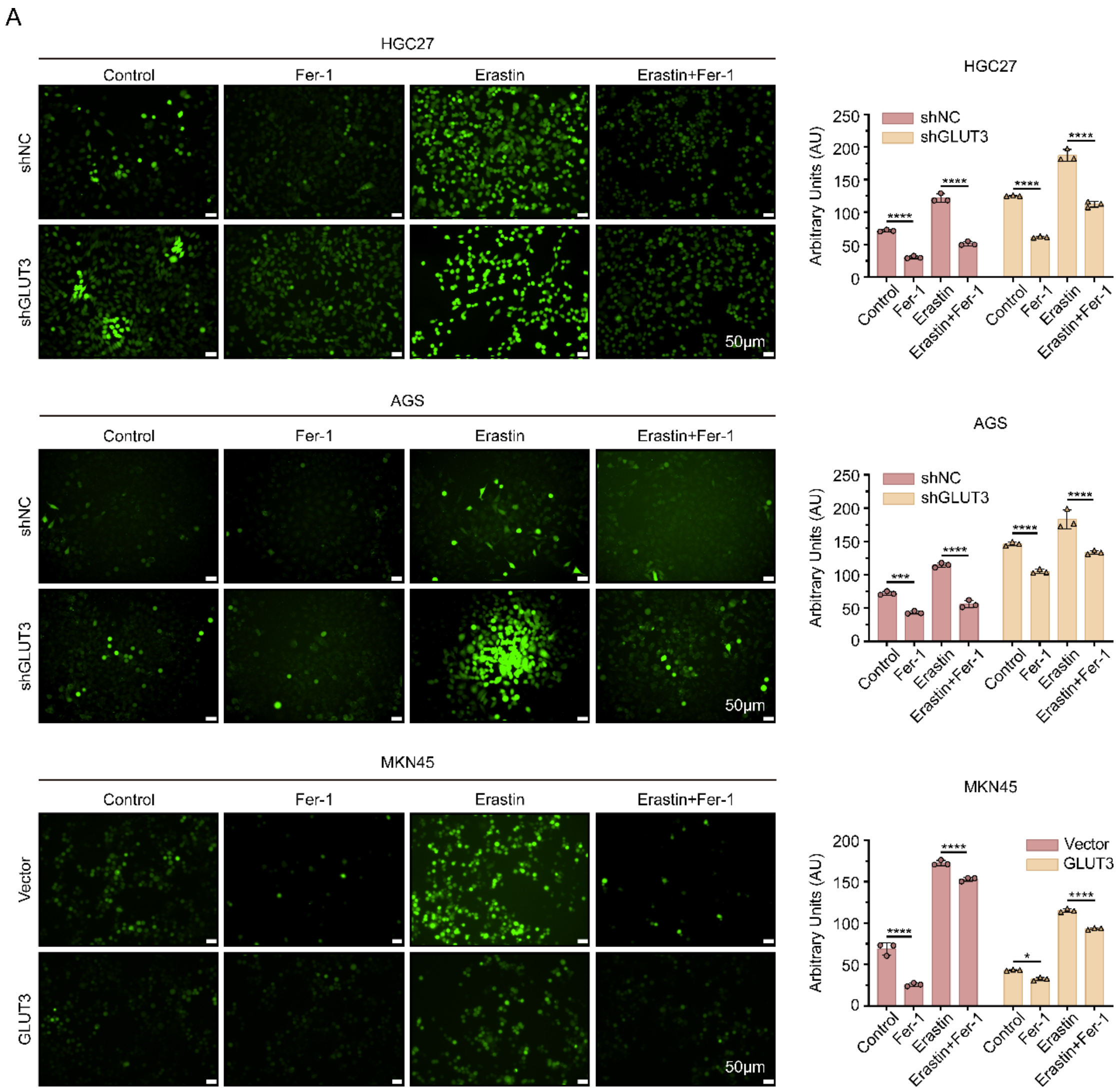

Figure S6. Total ROS detection in gastric cancer cells. A) AGS/shGLUT3, HGC27/shGLUT3 and MKN45/GLUT3 cells and their negative control cells were treated with or without Erastin (10 μM), Fer-1 (2 μM), and the Erastin/Fer-1 mixture for 24 h, then the cells were stained by DCFH-DA and observed under an inverted fluorescence microscope. (Green fluorescence: Total ROS detected by DCFH-DA probe; Scale bar: 50 μm.) Quantification of intracellular total ROS levels. All data are presented as the mean ± SD. *$P<0.05$*, ***$P<0.001$*, ****$P<0.0001$*. Abbreviations: ROS: Reactive Oxygen Species; Fer-1: Ferrostatin-1.

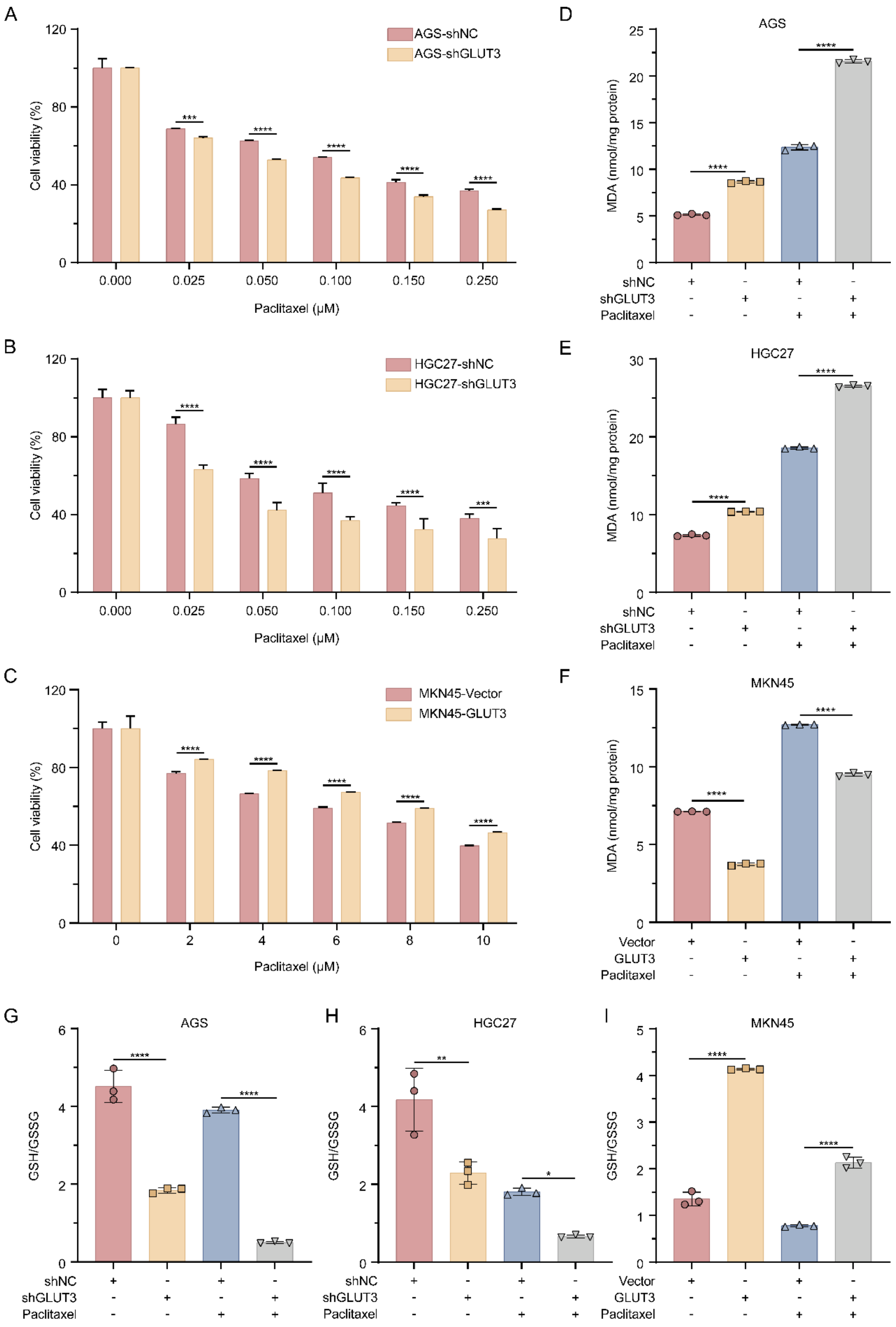

Figure S7. GLUT3 protects GC cells from paclitaxel-induced death by inhibiting ferroptosis. A-C) AGS/shGLUT3, HGC27/shGLUT3 and MKN45/GLUT3 cells and

their negative control cells were treated with increasing concentrations of paclitaxel (AGS/HGC27: 0, 0.025, 0.05, 0.1, 0.15 and 0.25 μM; MKN45: 0, 2, 4, 6, 8, 10 μM) for 24 h, and the viability of cells was measured using CCK8 assay. D-F) Levels of MDA in the indicated cells were measured upon treatment with or without paclitaxel (AGS/HGC27: 0.1 μM; MKN45: 10 μM) for 24 h. G-I) The ratio of GSH/GSSG in the indicated cells was measured upon treatment with or without paclitaxel (AGS/HGC27: 0.1 μM; MKN45: 10 μM) for 24 h. All data are presented as the mean ± SD. $^{*}P<0.05$, $^{**}P<0.01$, $^{***}P<0.001$, $^{****}P<0.0001$. Abbreviations: CCK8: Cell Counting Kit-8; MDA: Malondialdehyde; GSH: Glutathione (reduced); GSSG: Glutathione Disulfide (oxidized).

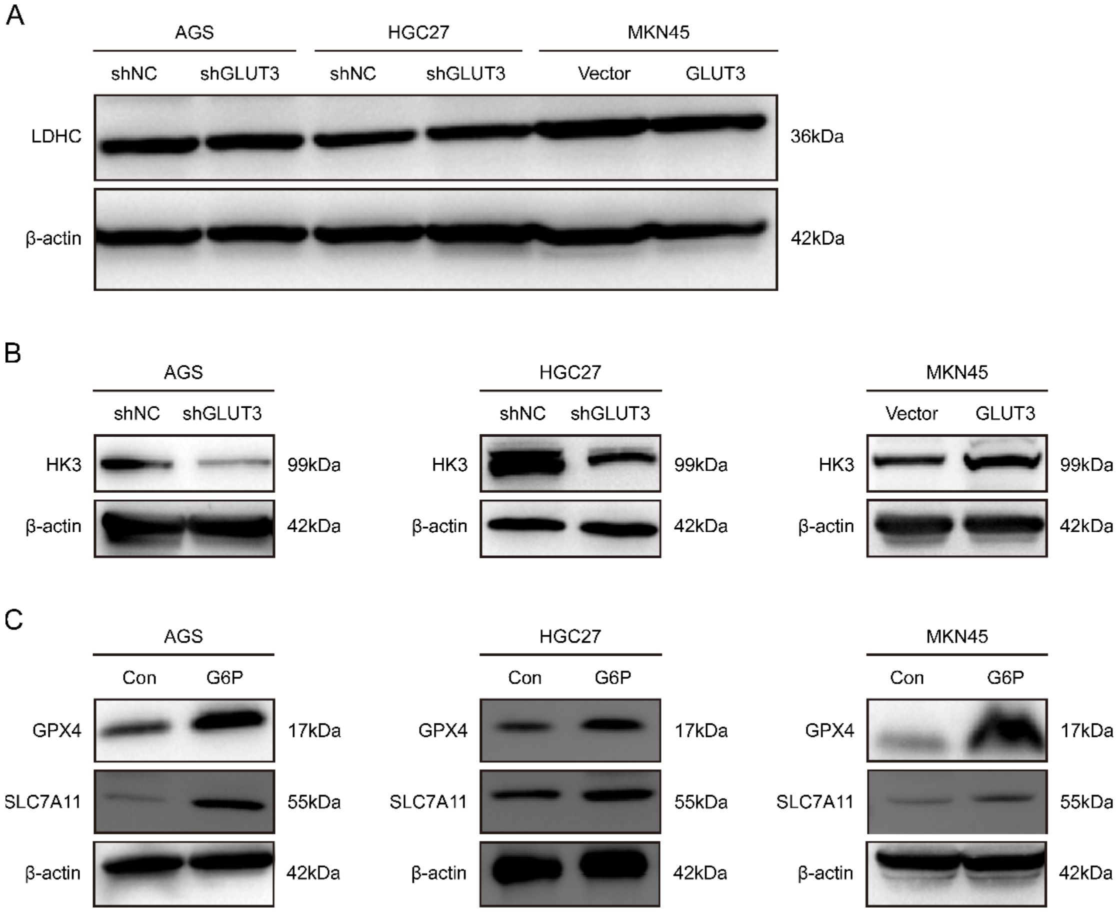

Figure S8. Protein expression of LDHC, HK3, GPX4, and SLC7A11 in gastric cancer cells. A) Protein expression of LDHC in AGS/shGLUT3, HGC27/shGLUT3, MKN45/GLUT3 cells and their negative control cells. B) Protein expression of HK3 in the indicated gastric cancer cells. C) Protein expression of GPX4 and SLC7A11 in gastric cancer cells with or without G6P stimulation (500 μM) for 24 h. Abbreviations: Con: Control; G6P: Glucose-6-phosphate.

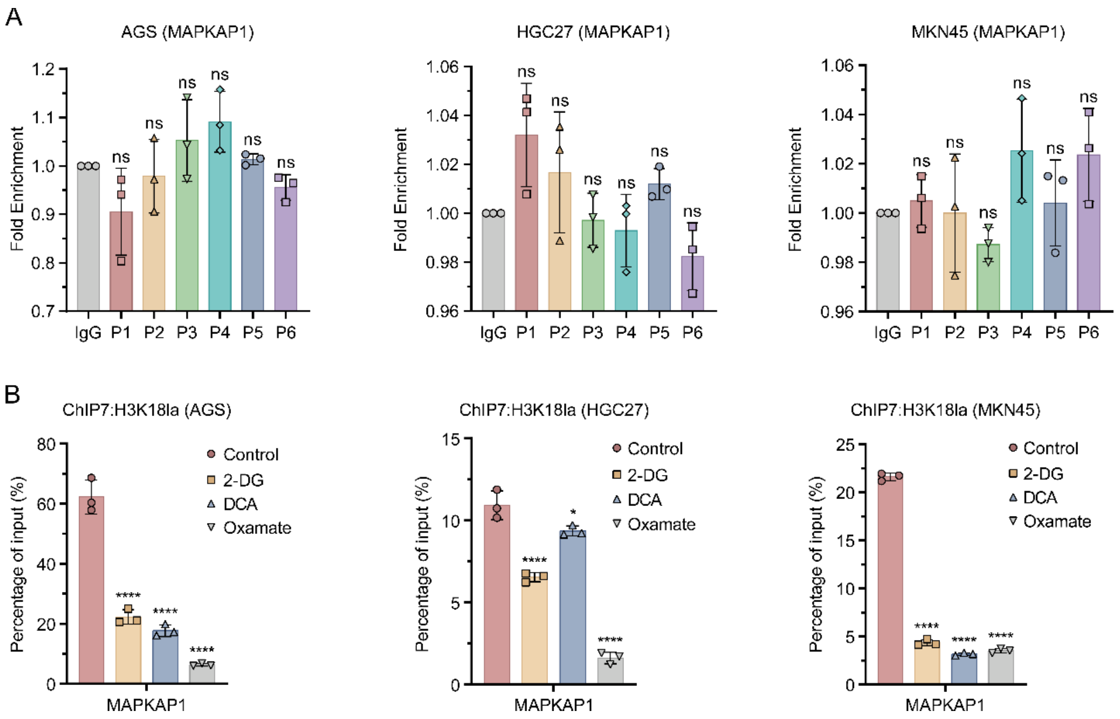

Figure S9. ChIP-qPCR analysis of region-specific H3K18la enrichment at the MAPKAP1 promoter and its modulation by glycolytic inhibitors in GC cells. A) ChIP-qPCR assay of H3K18la status in the MAPKAP1 promoter region 1-6 in AGS, HGC27 and MKN45 cells. B) Effect of glycolytic inhibitors 2-DG, Oxamate, or DCA on H3K18la enrichment at the MAPKAP1 promoter region 7 in wild-type gastric cancer cells, as determined by ChIP-qPCR and presented as percentage of input relative to untreated controls. Cells were treated with 10 mM 2-DG, 20 mM Oxamate, or 5 mM DCA for 48 hours. All data are presented as the mean ± SD. **P<0.05*, *****P<0.0001*, ns: not significant. Abbreviations: DCA: Dichloroacetate; 2-DG: 2-Deoxy-D-glucose.

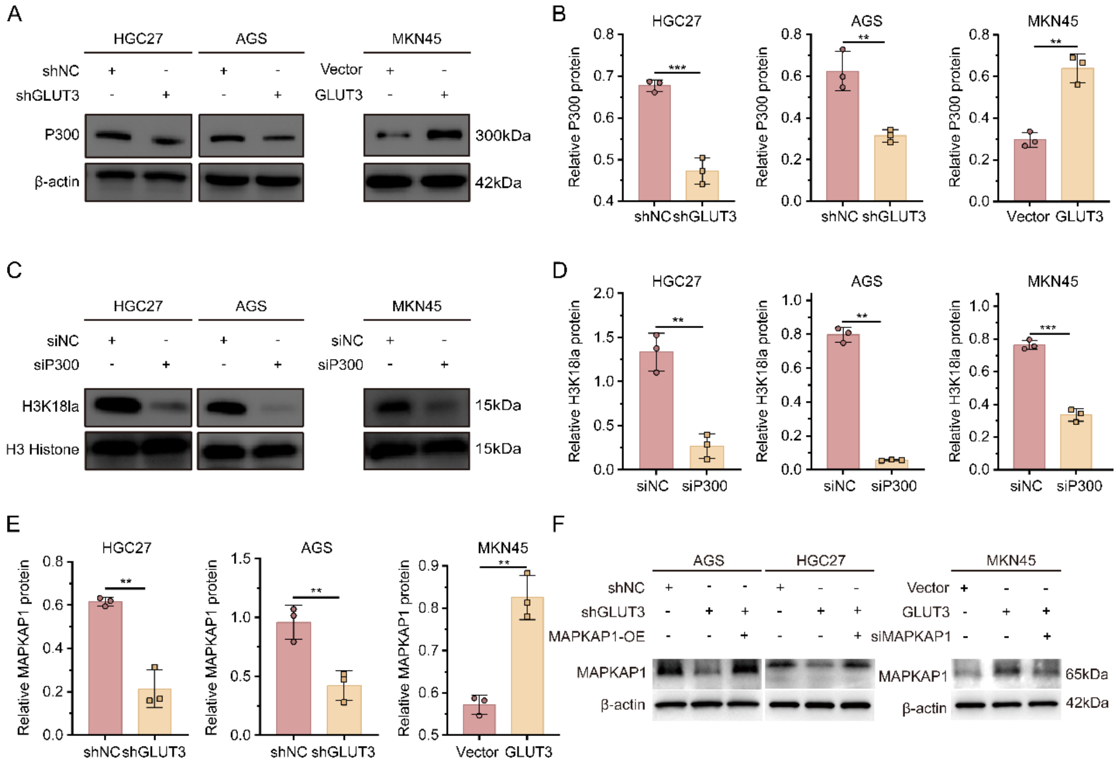

Figure S10. Protein expression analyses by Western blotting. A-B) Protein expression of P300 in AGS/shGLUT3, HGC27/shGLUT3, MKN45/GLUT3 cells and their negative control cells. C-D) Protein expression of H3K18la in AGS/siP300, HGC27/siP300, MKN45/siP300 cells and their negative control cells. E) Protein expression of MAPKAP1 in the indicated gastric cancer cells. F) Knockdown and overexpression efficiency of MAPKAP1 plasmid transfection in specific gastric cancer cells. All data are presented as the mean ± SD. $**P<0.01$, $***P<0.001$.

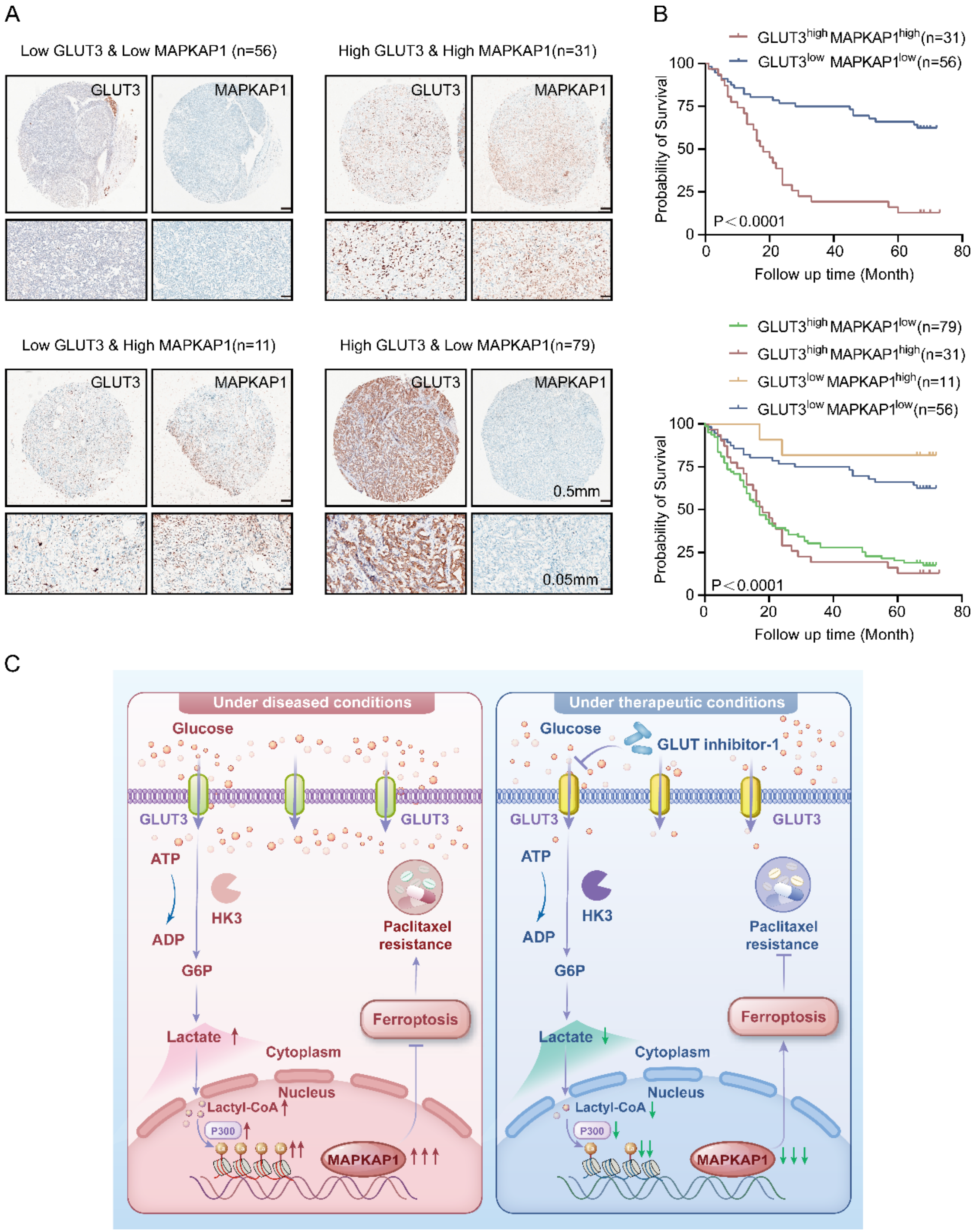

Figure S11. Concurrent high expression of GLUT3 and MAPKAP1 in GC tissues correlates with poor patient prognosis. A) Representative IHC staining of GLUT3 and MAPKAP1 expression patterns in the indicated groups are shown. Scale bar: 500 μm & 50 μm. B) Kaplan-Meier survival analysis of overall survival rate according to the expression of the combination of GLUT3 and MAPKAP1 in GC patients using log-

rank test. C) A schematic model of GLUT3 protects GC against ferroptosis by activating MAPKAP1. GLUT3 upregulates HK3 expression, increasing G6P and lactate production. P300 utilizes lactate-derived lactoyl-CoA as a substrate to catalyze lactylation modification at lysine 18 of histone H3 (H3K18la) within the promoter region of the MAPKAP1 gene. This epigenetic modification transcriptionally upregulates MAPKAP1, ultimately inhibiting ferroptosis and promoting the progression and paclitaxel-resistance of GC.
